# CRISPR/Cas9-mediated inactivation of the soybean agglutinin *Le1* gene to improve grain quality

**DOI:** 10.3389/fpls.2025.1737513

**Published:** 2026-01-09

**Authors:** João Matheus Kafer, Alessandra Koltun, Rodrigo Thibes Hoshino, Larissa Girotto, Cesar Augusto Silveira, Silvana Regina Rockenbach Marin, Elibio Leopoldo Rech, Alexandre Lima Nepomuceno, Liliane Marcia Mertz-Henning

**Affiliations:** 1 Embrapa Soja, Londrina, Paraná, Brazil; 2 Department of General Biology, State University of Londrina, Londrina, Paraná, Brazil; 3 Department of Agronomy, State University of Maringá, Maringá, Paraná, Brazil; 4 Fundação de Apoio à Pesquisa e ao Desenvolvimento (FAPED), Sete Lagoas, Minas Gerais, Brazil; 5 Conselho Nacional de Desenvolvimento Científico e Tecnológico (CNPq), Brasília, Distrito Federal, Brazil; 6 Embrapa Recursos Genéticos e Biotecnologia, Brasília, Distrito Federal, Brazil

**Keywords:** agglutinin, antinutritional factor, *Glycine max*, knockout, gene editing

## Abstract

**Introduction:**

Soybean agglutinin (SBA) is a major antinutritional factor in soybean seeds, reducing digestibility in monogastric animals. The Le1 gene encodes the primary lectin accumulated in seeds. Genome editing offers a direct strategy to eliminate this factor in elite cultivars.

**Methods:**

Two gRNAs targeting Le1 were inserted into a CRISPR/Cas9 binary vector and used for Agrobacterium tumefaciens–mediated transformation of the soybean cultivar BRS 537. Edited plants were screened by PCR, Sanger sequencing, protein electrophoresis (SDS-PAGE), hemagglutination assays, and segregating generations were tested to identify transgene-free progeny. Agronomic traits were evaluated under field conditions.

**Results:**

Twenty transformation events were generated, with an editing efficiency of 10%. Event AF12-13-1 carried a 4-bp deletion producing a truncated, unstable lectin protein. SDS-PAGE confirmed the absence of the ~30 kDa SBA band, and hemagglutination assays showed complete loss of lectin activity. Transgene-free T2 plants lacking Bar, Cas9, and AtU6 sequences were identified. Agronomic traits—including yield and thousand-seed weight—were comparable to the wild-type cultivar.

**Discussion:**

CRISPR/Cas9 editing of Le1 effectively eliminated SBA accumulation without compromising key agronomic traits. The resulting low-lectin soybean lines represent a promising approach to improve digestibility and feed efficiency for monogastric animals.

## 1 Introduction

Soybean (*Glycine max* Merril.) is a major legume crop, highly valued for its seeds, which are rich in protein (∼40%), oil (∼20%), and carbohydrates (26–30%) (George et al., 2008; Choi et al., 2022; Padalkar et al., 2023). Soybean and its derivatives are widely used across the food, feed, pharmaceutical, and industrial sectors (Singh et al., 2008; Rizzo et al., 2018). Although soybean meal represents a major global protein source for animal feed, soybean proteins also play important roles in human nutrition and functional food applications, owing to their physicochemical, bioactive, and technological properties (Messina, 2016). Even in human nutrition, soybean is regarded as a functional food, owing to its medicinal and health-promoting properties, which include a range of bioactive compounds such as isoflavones, flavonols, proteins, and lipids (Padalkar et al., 2023).

Despite its nutritional and medicinal value, soybean contains antinutritional factors that limit its use in both human consumption and animal feed, as they impair nutrient digestibility and absorption in the gastrointestinal tract. Among the many antinutritional factors, the most relevant are the Kunitz trypsin inhibitor (KTI) and soybean agglutinins, or lectins (Schmidt et al., 2015; Choi et al., 2022; Padalkar et al., 2023).

Structurally, lectins possess a characteristic domain and are classified according to their molecular structure and carbohydrate-binding properties. In plants, lectins perform different functions depending on their subcellular localization. Plant lectins can participate in defense by acting as signaling or recognition molecules in nucleocytoplasmic compartments, where they respond to biotic stresses (Chrispeels and Raikhel, 1991; Lannoo and Van Damme, 2014; De Coninck and Van Damme, 2022). In contrast, seed lectins, which accumulate predominantly in protein storage vacuoles, act mainly as storage proteins rather than components of signaling-driven defense mechanisms (De Coninck and Van Damme, 2021, 2022).

Ingestion of tissues from storage organs, such as seeds, releases lectins into the animal intestine, where they bind to glycan receptors on the intestinal wall. This lectin–carbohydrate interaction alters intestinal epithelial morphology and impairs nutrient digestion and absorption (Alves de Moraes et al., 2006; George et al., 2008; Schmidt et al., 2015). Raw soybean seeds contain 10–20 g.kg^−1^ of lectin, with concentrations above 7 g.kg^−1^ considered detrimental to digestibility and unsuitable for direct consumption (George et al., 2008), limiting soybean use both as a source of human food and in animal feed formulations, including its use as a forage crop (Padalkar et al., 2023). Processing methods can inactivate these antinutritional factors, but they are costly and may reduce the nutritional content of soybean. For these reasons, genetic inactivation of antinutritional factors has become an attractive strategy.

In soybean, four transcribed legume lectin genes have been identified: the vegetative *Le3* (SVL/LE3), the pseudogene *Le2*, the seed-specific *Le1* (soybean lectin, also known as phytohemagglutinin or agglutinin), and *Le4*. In *Arabidopsis thaliana*, histochemical analyses showed that the *Le2* promoter drives expression in all tissues, including seeds, but not roots. Comparing the *Le1* promoter with *Le3* and *Le4* promoters revealed that the *Le1* promoter contains a higher number of seed-specific motifs (Chragh et al., 2015; Shamimuzzaman and Vodkin, 2018). The *Le1* gene encodes soybean lectin, a 120 kDa glycoprotein composed of four similar 30 kDa subunits. In recessive lectin genotypes (*Le1*), a 3.5 kb insertion element (Tgm1) was identified, which prevents transcription and, consequently, lectin accumulation in seeds. Furthermore, removal of Tgm1 from the mutant allele restores the gene’s ability to be transcribed and expressed during seed development (Goldberg et al., 1983; Vodkin et al., 1983; Okamuro and Goldberg, 1992). Therefore, *Le1* is responsible for the accumulation of this antinutritional factor in seeds and represents a highly valued target for strategies aiming to reduce lectin content through genetic approaches.

Developing genotypes that do not produce lectin using genome editing technology offers advantages over conventional breeding, as it can be applied directly to elite cultivars without the need for crosses to fix the trait in the target genotype. Accordingly, the aim of this study is to reduce lectin content in soybean seeds by knocking out the *Le1* gene using CRISPR/Cas9 technology in an elite soybean variety adapted to tropical climates, aiming to improve digestibility mainly for monogastric animals.

## 2 Materials and methods

### 2.1 Prospection of the *Le1* gene in soybean and in silico characterization

The *Le1* gene sequence was retrieved from the Wm82.a2.v1 and Wm82.a4.v1 versions of the *Glycine max* reference genome, available on Phytozome. Orthologous and paralogous genes were identified based on amino acid sequences from the PF00139 family (true lectins with the exclusive Lectin_legB domain), also obtained from Phytozome v13. Proteins containing additional domains or belonging to the PF03388 family (putative lectins) were excluded. Sequences were aligned using MUSCLE (Edgar, 2004), and a phylogenetic tree was constructed with iTOL (Letunic and Bork, 2024). Physicochemical similarities among proteins were analyzed using Persephone (https://web.persephonesoft.com/). Gene expression was assessed via the RNA-Seq Atlas (https://soyatlas.venanciogroup.uenf.br/), with comparisons made between homologous genes.

### 2.2 CRISPR vector construction and soybean transformation

gRNAs targeting the *Le1* gene (Glyma.02G012600) were designed using CRISPRDirect (Naito et al., 2015), and the most specific guide with no predicted off-targets in the guide + PAM region was selected. The modular vector C034p7ioR-35SCasWT (DNA Cloning Service, Hamburg, Germany), which contains two *Bsm*BI sites for gRNA insertion, was employed. Cohesive ends for cloning were designed as follows: oligo 1 – 5’ TGATTG N(20) 3’ and oligo 2 – 5’ AAAC N(20) CA 3’, with an additional 5’ guanine to optimize transcription from the U6 promoter. The vector expresses Cas9 under the CaMV 35S promoter, the *Bar* gene conferring resistance to ammonium glufosinate, and the gRNA under the control of the *Arabidopsis thaliana* U6 promoter. Stable transformation of the soybean cultivar BRS 537 was conducted via Agrobacterium tumefaciens following Paz et al., 2005with minor modifications. Cotyledonary node explants from sterilized, germinated seeds were gently wounded with a sterile microbrush and infected with Agrobacterium cultures (OD_600_ ≈ 0.6). After a 5-day co-cultivation, explants were transferred to shoot induction and elongation media supplemented with glufosinate ammonium for selection. Regenerated shoots were rooted under selection, acclimated, and screened by PCR to confirm the presence of transgenic sequences.

### 2.3 Polymerase chain reaction for detection of transgenes

Genomic DNA was extracted from leaves using a modified CTAB method (Doyle and Doyle, 1987). DNA quality and concentration were assessed by spectrophotometry (260/280 nm) and 1% agarose gel electrophoresis. The presence of transgenic sequences was verified by conventional PCR using primers specific for the *Bar* and Cas9 genes, and the *At*U6 promoter. Amplified products were separated on 1.5% agarose gels containing ethidium bromide, visualized under a UV transilluminator, and documented by photodocumentation. The presence of bands at expected sizes was considered indicative of transgenesis. Primer sequences and the corresponding expected PCR product sizes are detailed in **Supplementary Table 1**.

### 2.4 Sanger sequencing

The *Le1* gene was amplified by polymerase chain reaction (PCR) using primers flanking the target region of interest. Reactions were carried out with previously extracted genomic DNA in a mixture containing appropriate buffer, magnesium chloride, deoxynucleotide triphosphates (dNTPs), primers, and a thermostable DNA polymerase. Amplification was performed in a thermocycler under the following conditions: initial denaturation at 94°C for 3 minutes, followed by 35 cycles of 94°C for 30 seconds, annealing at 58°C for 30 seconds, and extension at 72°C for 1 minute, with a final extension at 72°C for 5 minutes. The amplified product was verified by agarose gel electrophoresis and subsequently purified using ExoSAP-IT™ to remove residual primers and dNTPs. Sequencing was performed using the BigDye™ Terminator v3.1 kit, followed by purification via ethanol/EDTA precipitation. Samples were resuspended in Hi-Di™ formamide and subjected to capillary electrophoresis on an ABI Prism 3500 automated sequencer (Applied Biosystems). Electropherograms were analyzed using the ICE platform (Synthego).

### 2.5 *In silico* protein structure analysis

For in silico protein analysis, the wild-type and mutant *Le1* gene sequences were translated to generate the corresponding protein sequences. Both protein sequences were assessed using the ExPASy platform (https://www.expasy.org/) to evaluate protein stability (Gasteiger et al., 2005). Subsequently, protein structure was analyzed based on crystallographic data, and Ramachandran plots were generated using Swiss-Model (Waterhouse et al., 2024). Finally, carbohydrate-binding capacity of the protein was evaluated using PyMOL (PyMOL, 2025) with the SBF1 crystallographic structure as reference (Olsen et al., 1997).

### 2.6 Polyacrylamide gel electrophoresis analysis

Sample preparation for SDS-PAGE followed Alves de Moraes et al. (2006), with adaptations. Protein analysis was conducted by denaturing polyacrylamide gel electrophoresis in the presence of sodium dodecyl sulfate (SDS-PAGE), according to the method described by Laemmli (1970). The separation gel was prepared as a gradient (20–8% acrylamide) at pH 8.8, with a 4% stacking gel at pH 6.8. Electrophoresis was performed in Tris-glycine-SDS buffer (25 mM Tris, 192 mM glycine, 0.1% SDS, pH 8.3) at a constant voltage of 90 V until complete band separation. Gels were stained with Coomassie Brilliant Blue R-250 (0.1% in methanol:acetic acid:water, 45:10:45 v/v/v) for 1 hour and subsequently destained in methanol:acetic acid:water (30:10:60 v/v/v) until the background was cleared. Bands were visualized on a white light table, and molecular weights were estimated by comparison to a pre-stained protein marker.

### 2.7 Hemagglutination assay

The hemagglutination assay was performed according to (Adamcová et al., 2021), with adaptations. Human blood type A, containing the GalNAc carbohydrate for lectin detection, was used. Blood was collected from João Matheus Kafer and Human blood type A was collected using sodium citrate as anticoagulant following standard procedures (WHO, 2010). To isolate erythrocytes, 2 mL of human blood was centrifuged at 500 g, and the lymphocyte and plasma fractions were carefully removed using a Pasteur pipette. The blood was then washed three times with 0.1% PBS to prepare a 2% erythrocyte suspension in PBS. This suspension was treated with 0.05% trypsin freshly prepared in PBS, then washed three times with PBS for use in serial dilutions with the samples.

Sample preparation involved the grinding seeds from the AF12-13–1 plant, BRS 537 (background), and black bean (positive control), which were stored in a refrigerator until use. To extract soluble lectin, 5 g of each sample was diluted in 30 mL PBS and agitated at 250 RPM for 1 hour, followed by overnight storage at 4°C. Subsequently, 5 mL of the protein extract was centrifuged at 2850 g for 5 minutes, and the supernatant was recovered and centrifuged again at the same speed. This extract was used for preparing serial dilutions of the samples.

The AF12-13-1, BRS 537, and black bean samples underwent 10 serial dilutions (1/2, 1/4, 1/8, 1/16, 1/32, 1/64, 1/128, 1/256, 1/512, and 1/1024). Dilutions were distributed into reaction plates with an equal proportion of trypsin-treated erythrocytes in duplicate. Hemagglutination was observed visually, with a magnifying glass, and under a microscope. Hemagglutination was characterized by deformation of erythrocytes, exhibiting irregular shapes, whereas absence of hemagglutination was indicated by erythrocytes precipitating as a dot at the bottom of the plate.

### 2.8 Evaluation of agronomic traits

To evaluate agronomic traits, field trials were conducted at the EMBRAPA headquarters (Rodovia Carlos João Strass, s/n°, Acesso Orlando Amaral, Distrito da Warta; Coordinates: 23°11’6”S, 51°10’32”W). Plants were grown under standard field conditions with appropriate fertilization. The experiment was arranged in a randomized block design (DBC) with four replications, and each plot had an area of 8 m².

The evaluated traits included plant height (PH), thousand-seed weight (TSW), and productivity (PROD). PH was measured from the soil surface to the apex at physiological maturity. TSW was obtained from the mass of 1000 dried seeds. Productivity (PROD) was expressed as grain yield in kg·ha^−1^, calculated from the total seed weight harvested per plot and normalized to planting density. Oil and protein contents were quantified using Fourier transform near-infrared spectroscopy (FT-NIR; Antaris II, ThermoFisher Scientific, Waltham, MA, USA), following the protocol described by Mertz-Henning et al. (2017).

### 2.9 Statistical analysis

Statistical analyses were performed using RStudio version 4.4.3 (RStudio, 2020). Tukey’s test was applied at a 5% significance level.

## 3 Results

### 3.1 Identification of the *Le1* gene in soybean

**Figure 1:**
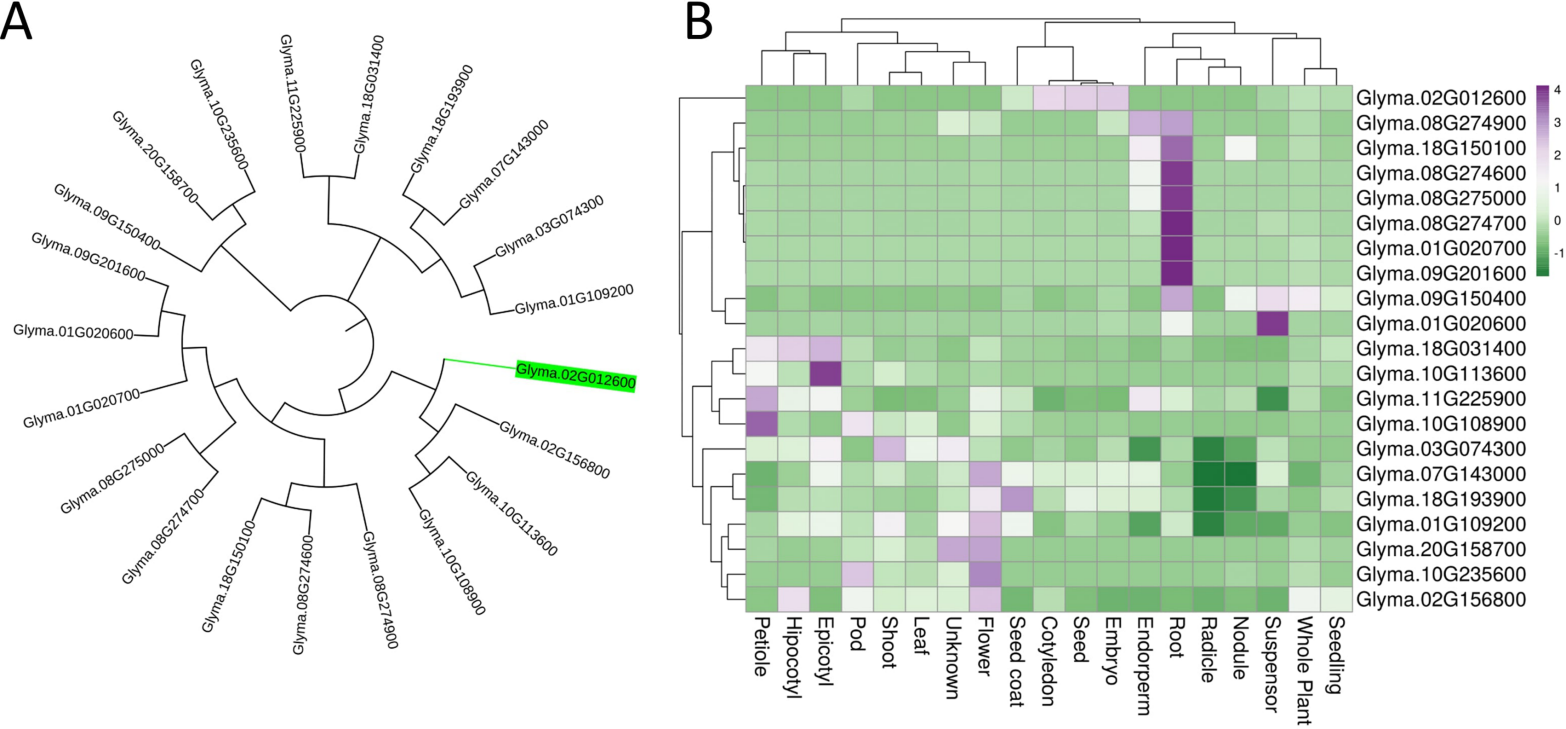
Characterization of soybean lectin genes from the PF00139 family. **(A)** Phylogenetic tree based on amino acid sequences. **(B)** Heatmap showing the average expression levels of PF00139 soybean lectin genes across different tissues.

Based on the *in silico* characterization of genes from the PF00139 family – which comprises the true lectins containing the Lectin_legB domain – 19 genes were identified as encoding true lectins with this single domain, distributed across 10 chromosomes (**Figure 1A**). Gene expression data from the Soybean Expression Atlas (https://soyatlas.venanciogroup.uenf.br/), using genome version Wm82.a4.v1, revealed their expression patterns across various tissues. The *Le1* gene (Glyma.02G012600), which encodes soybean seed agglutinin, is expressed mainly in the embryo derived tissues (**Figure 1B**). The gene most closely related to *Le1*, Glyma.02G156800, shares 62% structural similarity, but shows no detectable expression in seed tissues, as its expression is restricted to flowers and hypocotyls. These results suggest that *Le1* has a seed tissue-specific function and occurs as a single-copy gene.

### 3.2 Development of the *Le1* gene knockout vector

**Figure 2:**
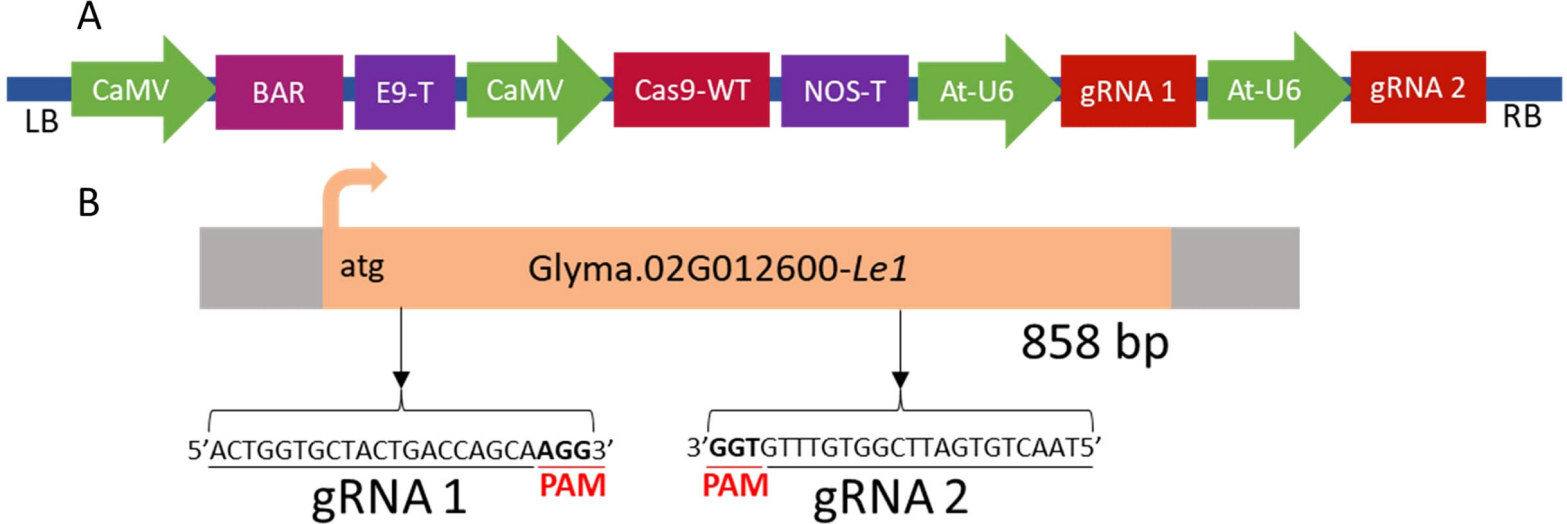
Vector map used for CRISPR/Cas9-mediated editing of the *Le1* gene **(A)**. gRNA target sites within *Le1***(B)**.

Vector cloning was performed following the protocol described by Koltun et al. (2023). The gRNAs were inserted into the modular vector C034p7ioR-35SCasWToi using type IIS restriction enzymes (*Bsm*BI). Cas9 nuclease expression was driven by the CaMV promoter. The Bar gene, under the control of the CaMV 35S promoter, was used to select transformed explants by conferring resistance to the herbicide glufosinate ammonium (**Figure 2A**). The vector includes two gRNAs targeting *Le1* gene sequence, as shown in **Figure 2B**.

Insertion of the gRNAs was confirmed by PCR using one primer annealing to the AtU6 promoter and another complementary to the gRNA sequence. Additionally, the PCR products were validated by Sanger sequencing. The vector was then transformed into *Agrobacterium tumefaciens* for use in soybean stable transformation.

### 3.3 Plant transformation and gene editing

**Figure 3:**
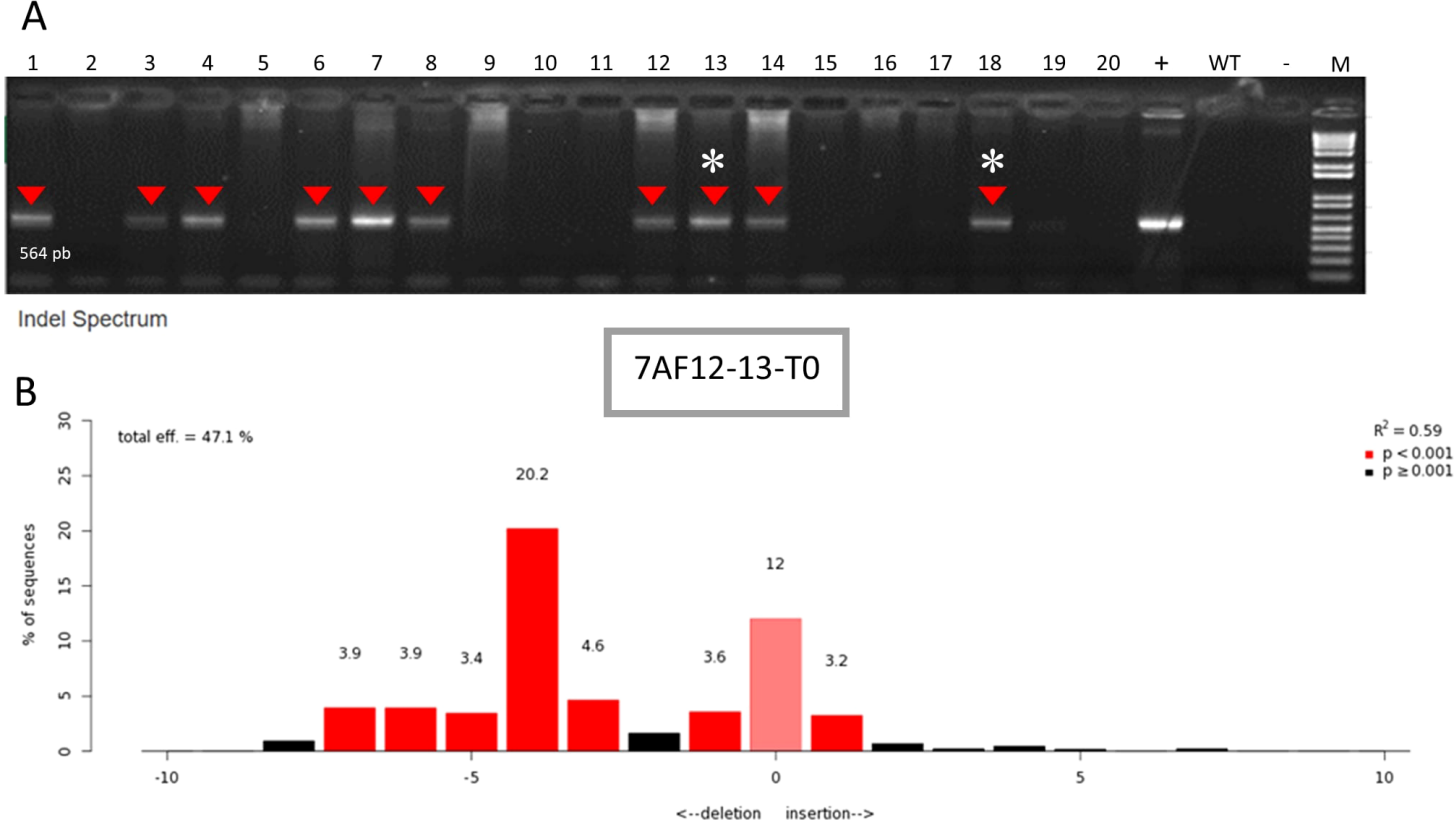
PCR detection of transformation plants confirming the presence of the CRISPR/Cas machinery using a primer annealing to the U6 promoter region **(A)**. Red arrows indicated positive transgenic plants; (*) indicate edited plants; (+) positive control; (WT) wild type; (–) negative control. Sanger sequencing of event AF12–13 analyzed with TIDE software to confirm *Le1* gene editing. **(B)** Target sites of gRNA1 and gRNA2 within the Le1 gene. The schematic representation shows the exon–intron structure and the relative positions of the two CRISPR/Cas9 cleavage sites, including the PAM sequences. Both gRNAs target exon regions to promote a frameshift-inducing deletion in the coding sequence of Le1.

A total of 285 soybean cotyledons were transformed via *Agrobacterium tumefaciens.* Of these, 20 putative transformant plants regenerated under selection and were transferred to acclimatization, where they completed their life cycle. Among them, 10 were confirmed as successfully transformed by PCR, using primers that detect the presence of the vector through amplification of the U6 promoter region, generating a 564 bp fragment (**Figure 3A**). Positive events were sequenced at the target gene region to confirm successful gene edition. Editing of the *Le1* gene was confirmed in two events, although only one of the two gRNAs was effective. Plant AF12-13, which showed the highest editing frequency, was selected for generation advancement (**Figure 3B**).

Based on sequence analysis using the TIDE software, the editing frequency in the T0 plant was 47.1%. Detected mutations ranged from –7 to +1 base pairs at the Cas9 cleavage site, with the most frequent mutation being a 4-bp deletion, accounting for 20.2% of all observed edits (**Figure 3B**). These results indicate transformation and editing efficiencies of 7.02% and 10%, respectively.

### 3.4 Editing and confirmation of mutants

**Figure 4:**
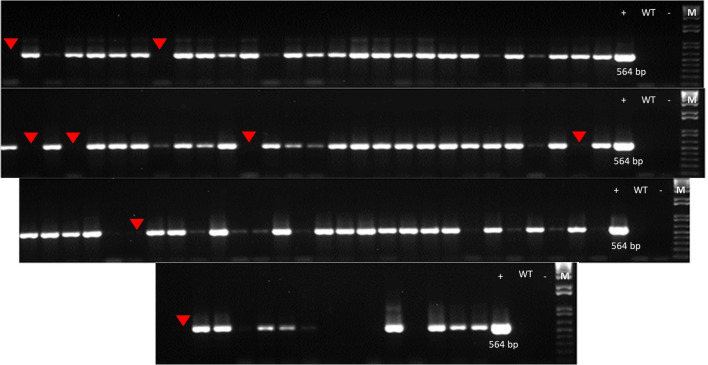
PCR amplification of fragments used for transgene detection in event AF12-13. Red arrows indicate T1 generation plants testing negative for the vector fragment.

Plant AF12-13 (T0) produced a total of 93 T1 seeds, which were planted in the greenhouse to advance generation and identify stable plants. T1 segregating plants were screened for the presence of the transgenic construct to identify edited plants free of exogenous DNA. Eight plants lacking the 564 bp vector fragment – detected using a primer annealing to the U6 promoter – were identified, five of them were selected for further confirmation (**Figure 4**).

**Figure 5:**
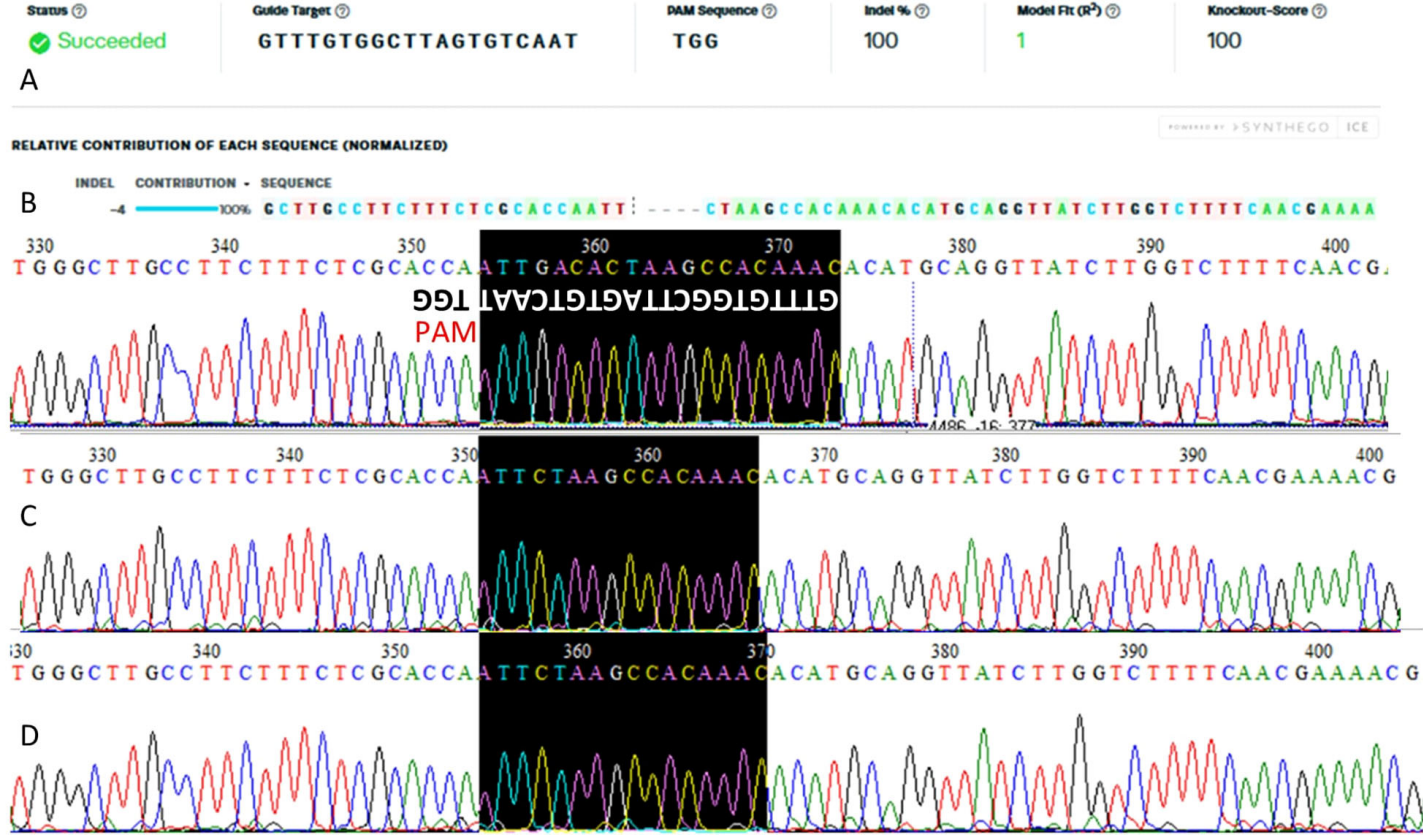
Sanger sequencing of events analyzed after the T0 generation, processed with ICE Synthego software **(A)**. Electropherogram of the wild-type plant highlighting the gRNA and PAM sequences **(B)**. Sequencing of T1 event AF12-13-1 **(C)** and T2 event AF12-13-1 **(D)**.

**Figure 6:**
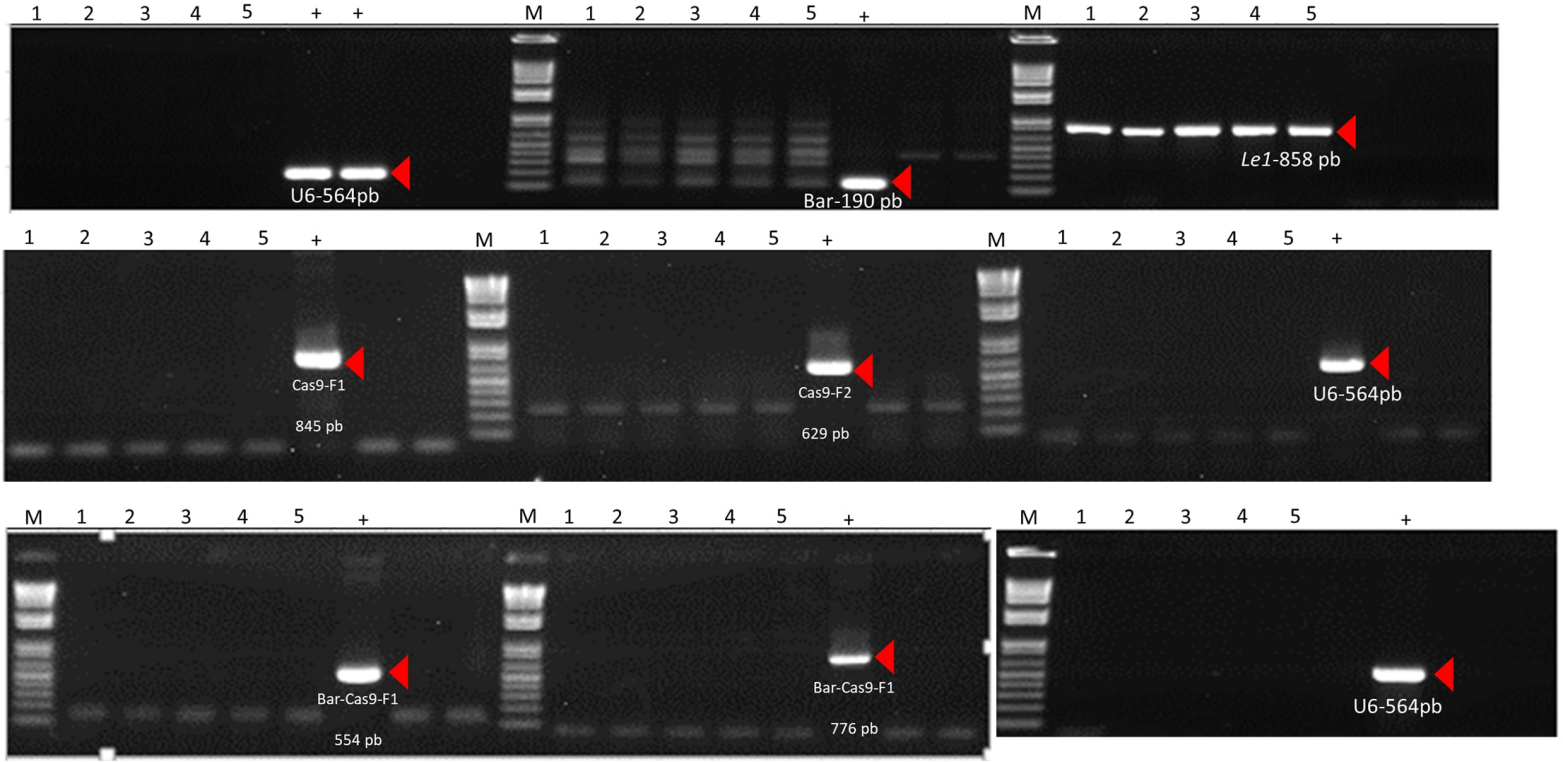
PCR amplification of fragments from DNA of 5 plants from event AF12-13. The empty vector was used as a positive control for the reaction, and amplification of the Le1 gene served as a DNA quality control (+). Red arrows indicate fragments amplified in the positive control (+).

Plants considered transgene-free, as they showed no presence of the vector, were selected for sequencing. Sanger sequencing revealed fixation of a 4-bp deletion (GACA) at the guide RNA target site in T1 plant AF12-13-1 (**Figures 5A–C**). A double verification was performed via PCR using primers amplifying different regions of the vector (U6 promoter, Cas9 nuclease, and Bar gene) (**Figure 6**) to confirm the absence of the CRISPR machinery. This plant was advanced to the next generation, and the mutation was confirmed in the T2 generation (**Figure 5D**). Sequencing of T2 AF12–13 plants confirmed that the 4-nucleotide deletion (GACA) at positions 388–391 of the *Le1* gene was stably inherited.

### 3.5 *In silico* characterization of mutants

**Figure 7:**
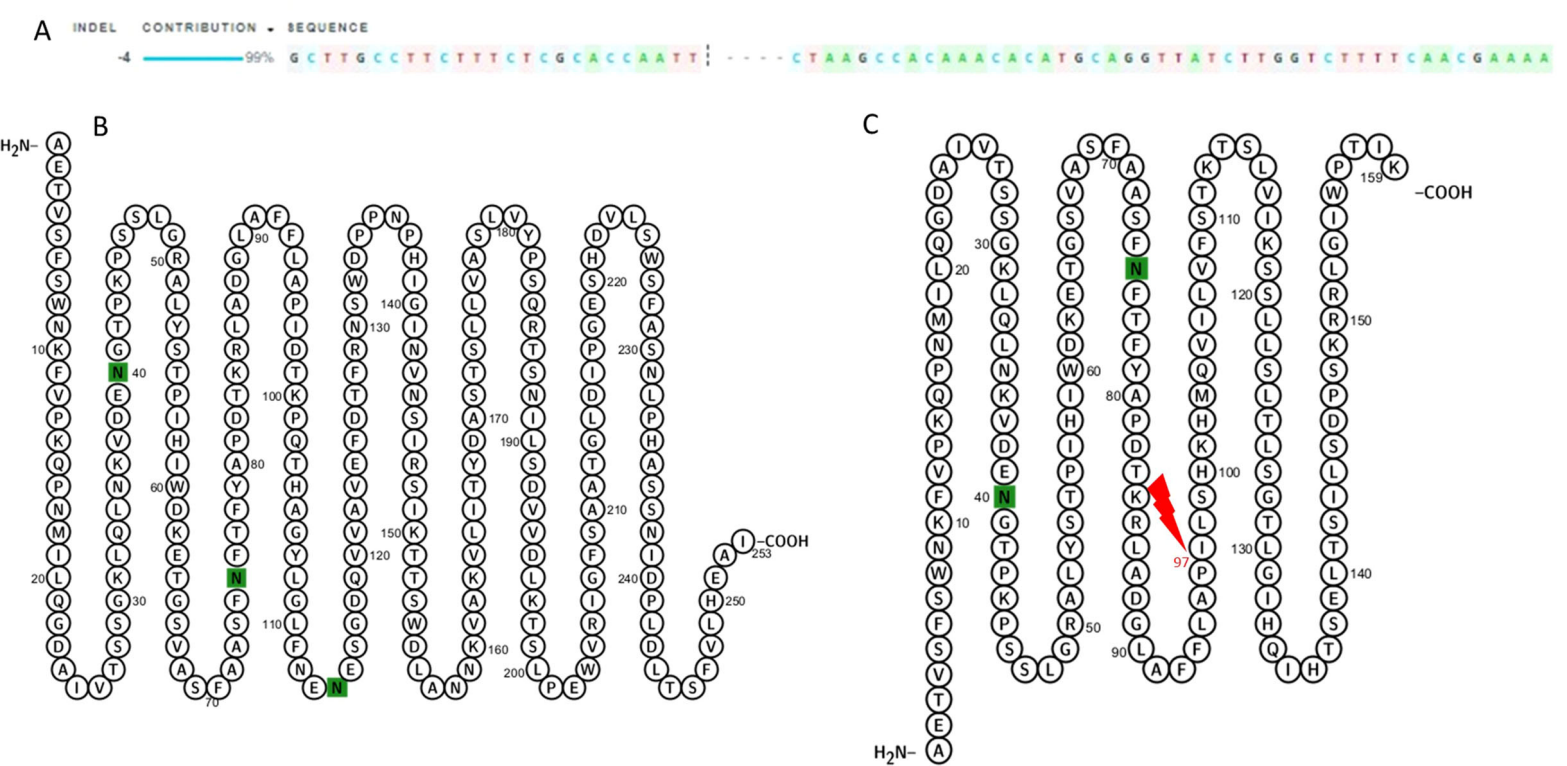
Comparison of *Le1* sequences between the BRS 537 wild type and genotype AF12-13-1. **(A)** Sanger sequencing of the *Le1* gene in the AF12-13–1 genotype. **(B)** Amino acid sequence of the *Le1* protein in the wild type. **(C)** Amino acid sequence of the *Le1* protein in the AF12-13–1 genotype.

The effects of the deletion were analyzed using the Muscle tool (https://www.ebi.ac.uk/Tools/msa/muscle/), which revealed a frameshift starting at the mutation site (**Figure 6C**). This frameshift introduced a premature stop codon, reducing the total protein length from 286 to 191 amino acids, including the 32-amino-acid signal peptide (**Figure 7**). Excluding the signal peptide, the wild-type protein contains 253 amino acids, whereas the mutated protein comprises 159 amino acids. This change results in protein instability, as indicated by ProtParam analysis (https://web.expasy.org/protparam/), which showed an increase in the instability index from 34.75 to 41.21, classifying the mutated protein as unstable (Guruprasad et al., 1990; Gasteiger et al., 2005).

Consequently, the molecular weight of the mutated protein decreased to 17.4 kDa, compared to 30 kDa in the wild type. The composition of charged residues also changed: the mutated protein contains 10 negatively charged and 16 positively charged amino acids, whereas the wild-type protein has 26 negatively and 21 positively charged residues. Both proteins share an identical amino acid sequence up to position 97.

**Figure 8:**
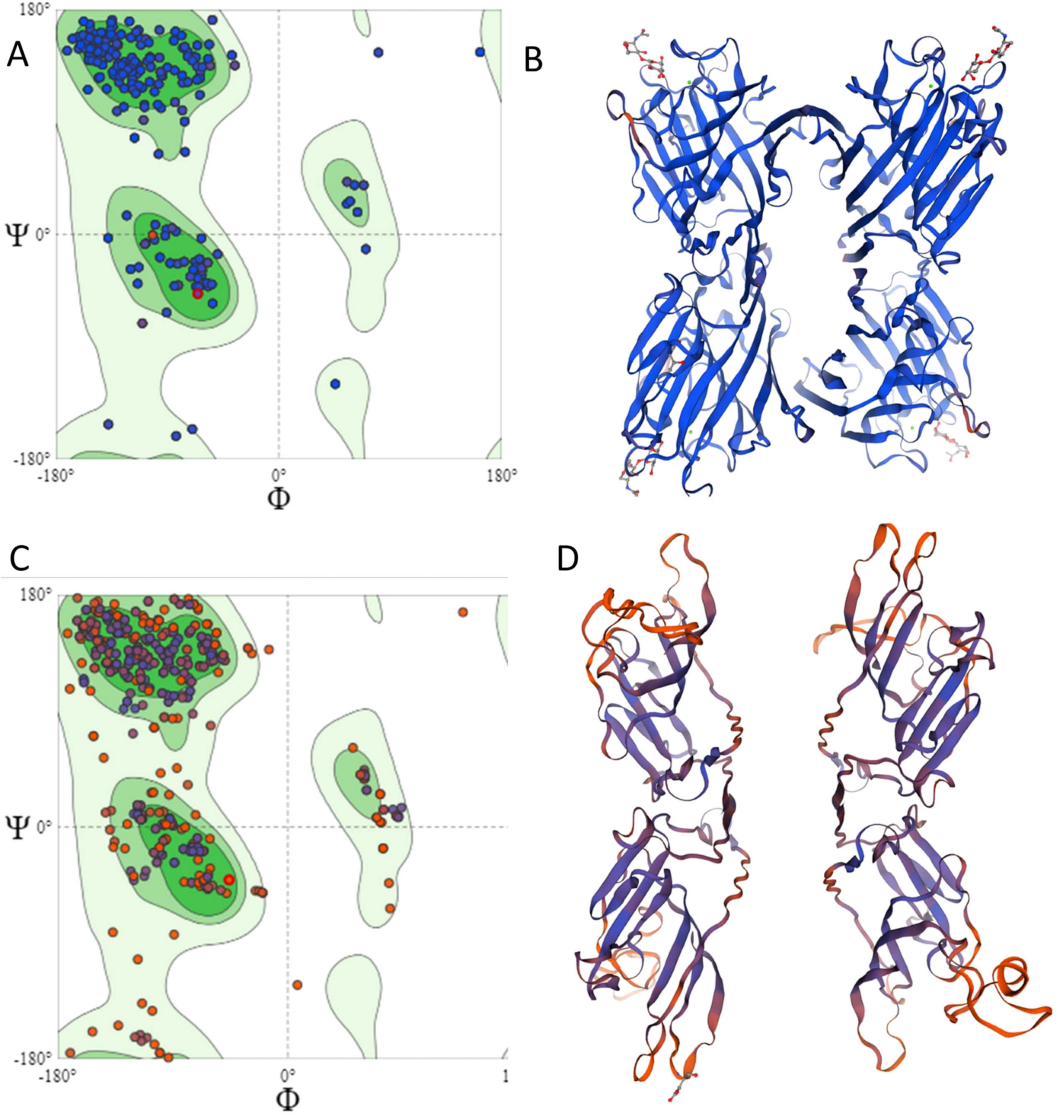
Structural comparison between the wild-type agglutinin and the mutated protein of AF12-13-1. **(A)** Ramachandran plot of the wild-type protein. **(B)** Crystallographic structure of the wild-type soybean agglutinin protein. **(C)** Ramachandran plot of the mutated protein. **(D)** Predicted crystallographic structure of the mutated protein from genotype AF12-13-1.

Using the Swiss-Model prediction tool, the mutated protein exhibited 43.17% identity with the proposed model, corresponding to the bean phytohemagglutinin. This divergence is likely due to the frameshift starting at amino acid 97. The effect is further illustrated in the Ramachandran plot, which shows the torsion angles of amino acids and their suitability for forming secondary and tertiary structures (**Figure 8**).

In **Figures 8A, B**, the wild-type protein structure shows a significant clustering of points around ϕ ≈ −60° and ψ ≈ −50°, indicating amino acids in an α-helix conformation. In **Figures 8C, D**, the mutated protein displays more residues outside this region, suggesting lower protein stability. Likewise, in the β-sheet region between ϕ ≈ −120° to −150° and ψ ≈ +100° to +150°, the mutated protein exhibits greater dispersion of points. Additionally, the mutated protein has a higher number of amino acids outside allowed regions, indicating reduced conformational stability.

**Figure 9:**
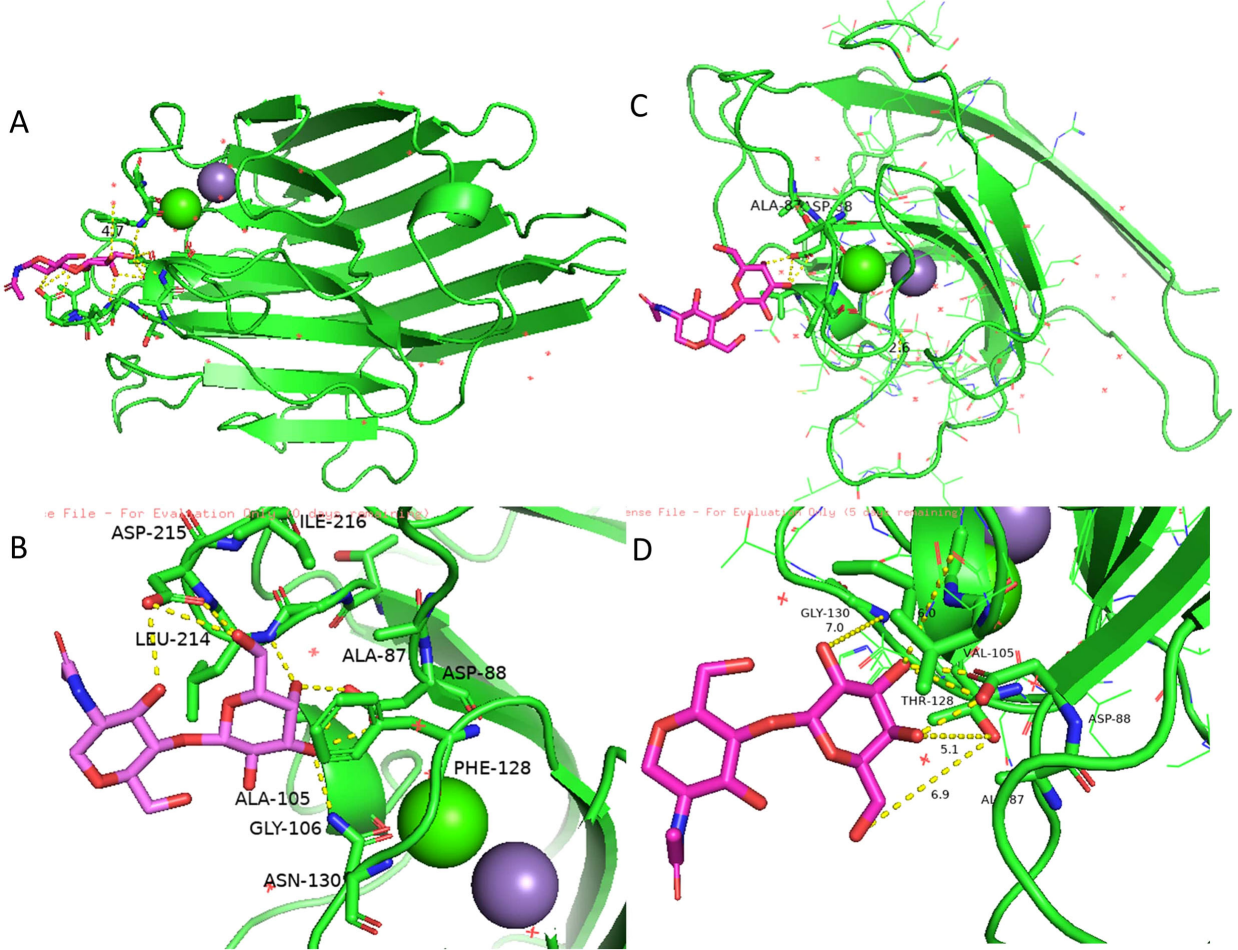
Structural model of the wild-type Le1 protein compared to the mutated protein. **(A)** Protomer of the wild-type protein from cultivar BRS 537. **(B)** Interactions with N-acetylglucosamine carbohydrate in the wild-type protein. **(C)** Protomer of the AF12-13–1 Le1. **(D)** Interactions with Nacetylglucosamine carbohydrate in the protein from AF12-13-1.

One of the main features of SBA as a lectin is its specific and reversible binding to carbohydrates, classifying it as a glycoprotein. Glycosylation enhances protein stability and ensures proper function. Therefore, we evaluated event AF12-13–1 based on its carbohydrate-binding capacity. SBA binds to the carbohydrate GalNAc through amino acids Ala-86, Asp-88, Ala-105, Phe-128, Asn-130, Leu-214, Asp-215, and Ile-216 (**Figure 9A**). The mutated protein from plant AF12-13–1 shows significant changes in the carbohydrate-binding site. While Ala-86 and Asp-88 remain, Ala-105, Phe-128, and Asn-130 are replaced by Val, Thr, and Gly, respectively. Additionally, Leu-214, Asp-215, and Ile-216 are absent due to the premature stop codon at position 191 (**Figure 9B**).

### 3.6 Denaturing protein gel analysis

**Figure 10:**
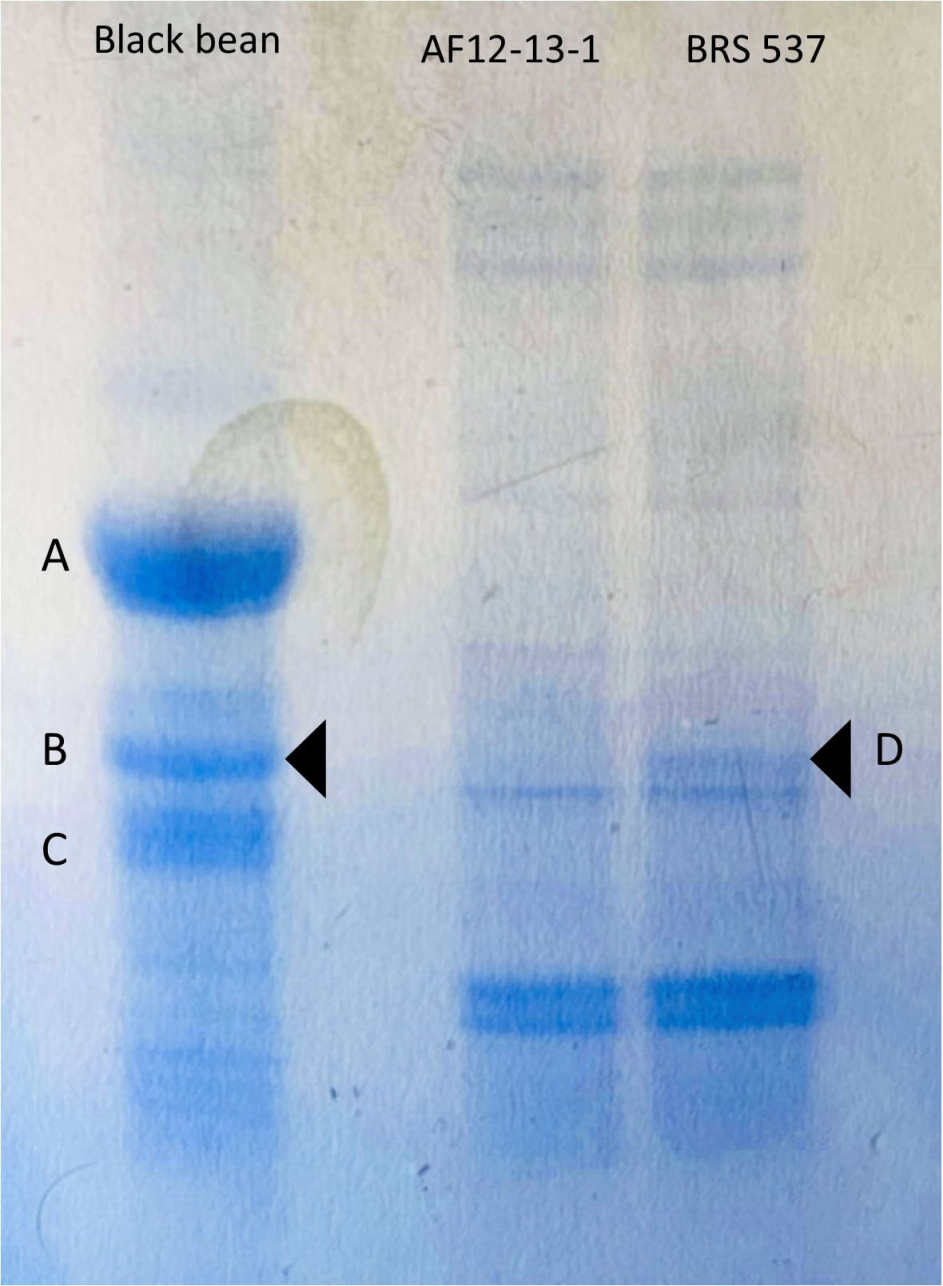
Polyacrylamide gel (SDS-PAGE) using protein extracts from genotypes BRS 537, AF12-13-1, and black bean as control. **(A)** 47 kDa Phaseolin (α-type). **(B)** 31 kDa PHA-E and PHA-L (bean lectins). **(C)** 25 kDa Phaseolin (β-type). **(D)** 30 kDa soybean agglutinin, with the band absent in genotype AF12-13-1.

To verify the presence of the protein in plant AF12-13-1, protein extracts were prepared from the BRS 537 (wild type) and AF12-13–1 genotypes, using black bean as a control. The results are shown in **Figure 10**: both the black bean and the wild-type BRS 537 genotype displayed a 30 kDa band corresponding to the lectin, while this band was absent in the AF12-13–1 genotype. This demonstrates that the frameshift mutation at positions 388–391 reduced the protein size.

### 3.7 Hemagglutination assay

**Figure 11:**
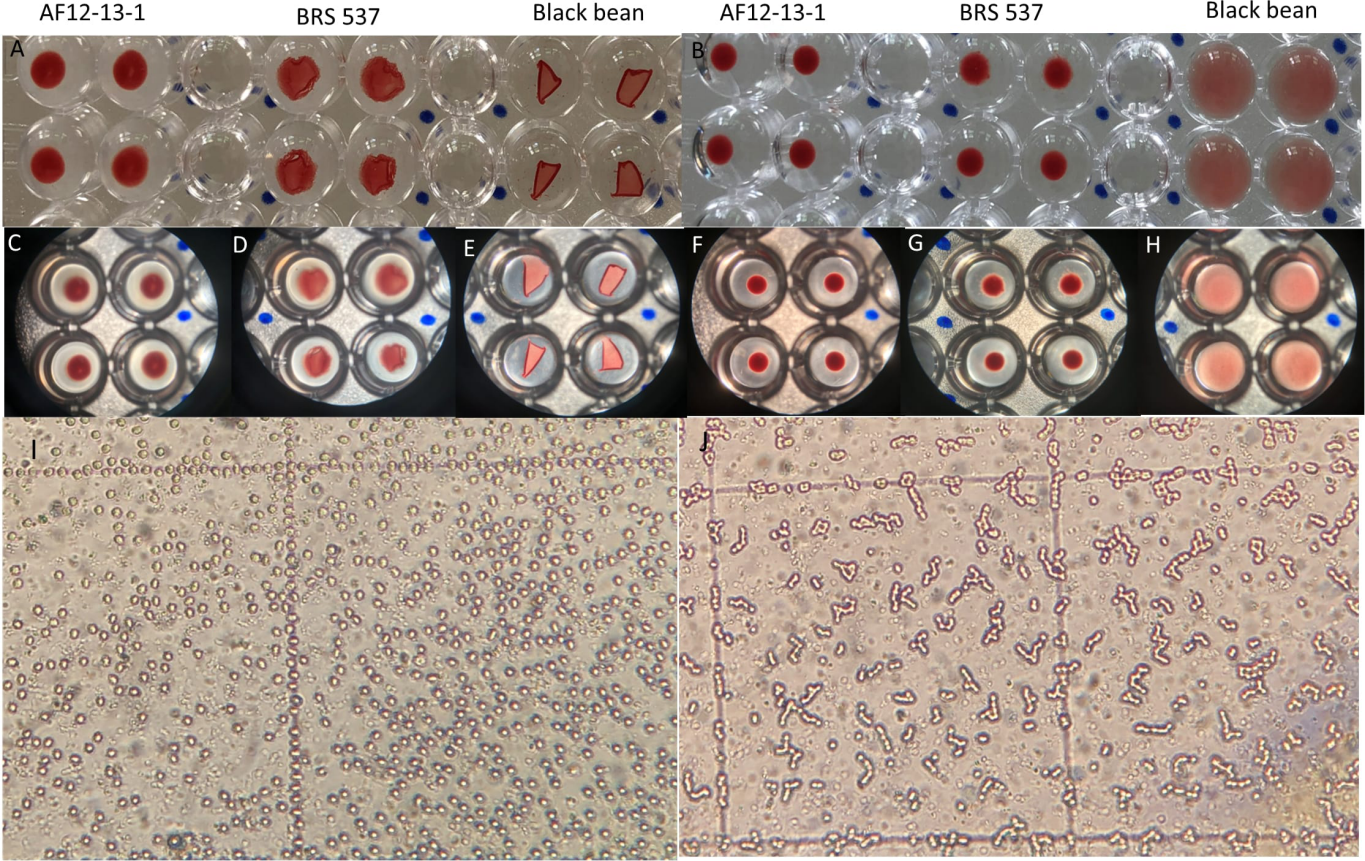
Hemagglutination analysis of protein extracts from soybean genotypes AF12-13-1 (edited genotype), BRS 537 (wild type), and Black Bean (positive control). **(A)** Naked-eye view of microplate wells at 1/16 and 1/32 dilutions. **(B)** Naked-eye view of microplate wells at 1/512 and 1/1024 dilutions. **(C–E)** Magnified microplate views at 1/16 and 1/32 dilutions for genotypes AF12-13-1, BRS 537, and Black Bean, respectively. **(F–H)** Magnified microplate views at 1/512 and 1/1024 dilutions for genotypes AF12-13-1, BRS 537, and Black Bean, respectively. **(I)** Edited genotype and **(J)** wild type: microscope views at 1/32 dilution.

To assess the biological activity of SBA in carbohydrate binding and quaternary structure formation, a hemagglutination test was performed using event AF12-13-1, the wild-type cultivar BRS 537, and black bean as a positive control. Human blood type A, which contains the SBA-binding carbohydrate GalNAc, was used. In **Figure 11**, the absence of hemagglutination is observed for plant AF12-13–1 at all dilutions, demonstrating that the protein does not form the quaternary structure.

### 3.8 Agronomic evaluation

**Figure 12:**
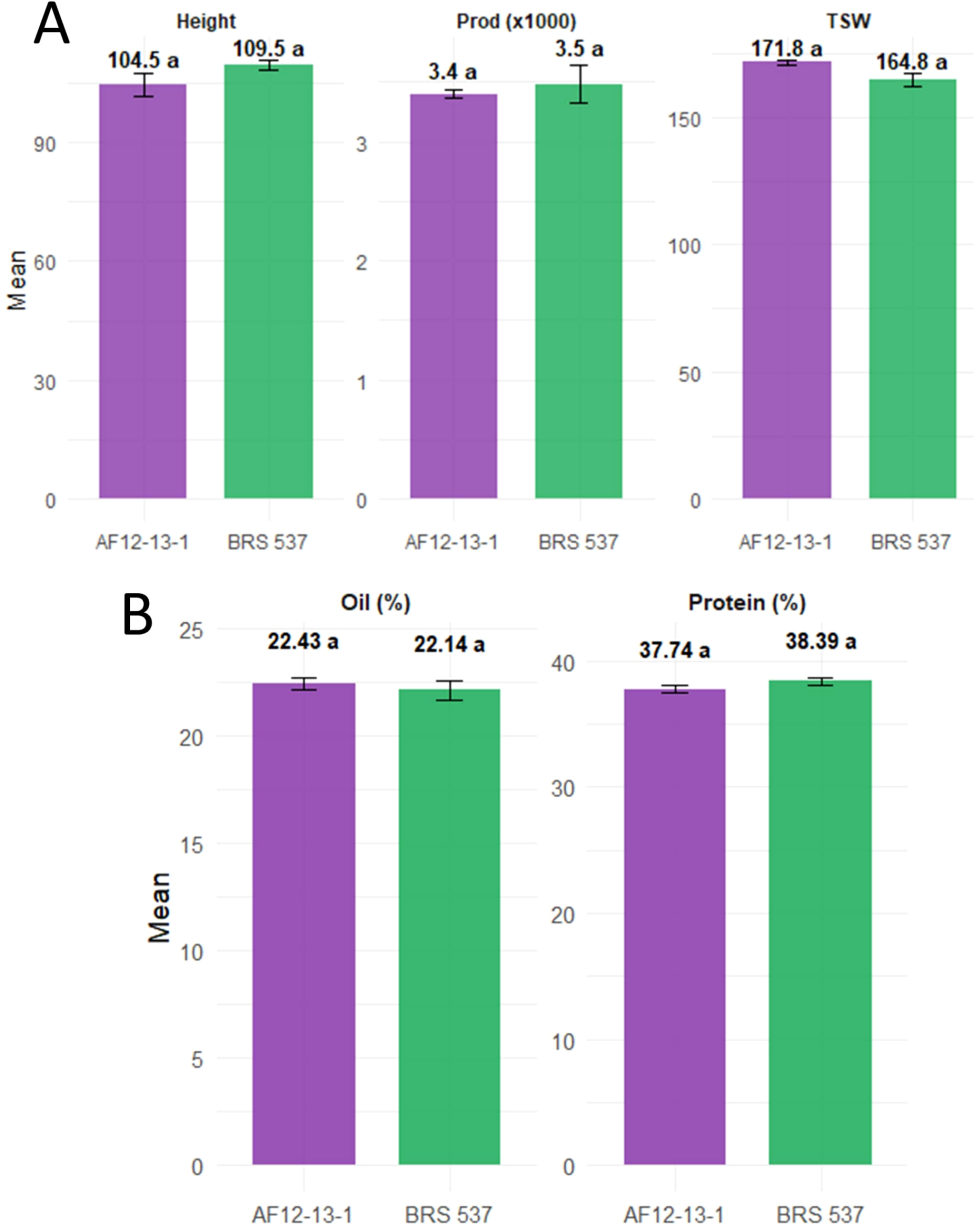
Agronomical performance of the genotypes AF12-13–1 and BRS 537 for the variables. **(A)** height (PH), productivity (PROD), thousand-seed weight (TSW). **(B)** Protein (%) and Oil (%). Different letters indicate significant differences between genotypes AF12-13–1 and BRS 537 at a 5% error probability according to Tukey’s test.

For the agronomic traits evaluated, including plant height (PH), thousand-seed weight (TSW) and productivity (PROD), no significant differences were observed between the edited genotype and the wild-type cultivar (**Figure 12**). PH was measured at physiological maturity, TSW was obtained from the mass of 1000 dried seeds, and PROD was calculated as grain yield per plant under field conditions. Similarly, protein and oil contents showed no significant variation between genotypes. Overall, the edited line displayed agronomic performance comparable to the non-edited cultivar, with only minor differences.

## 4 Discussion

**Figure 13:**
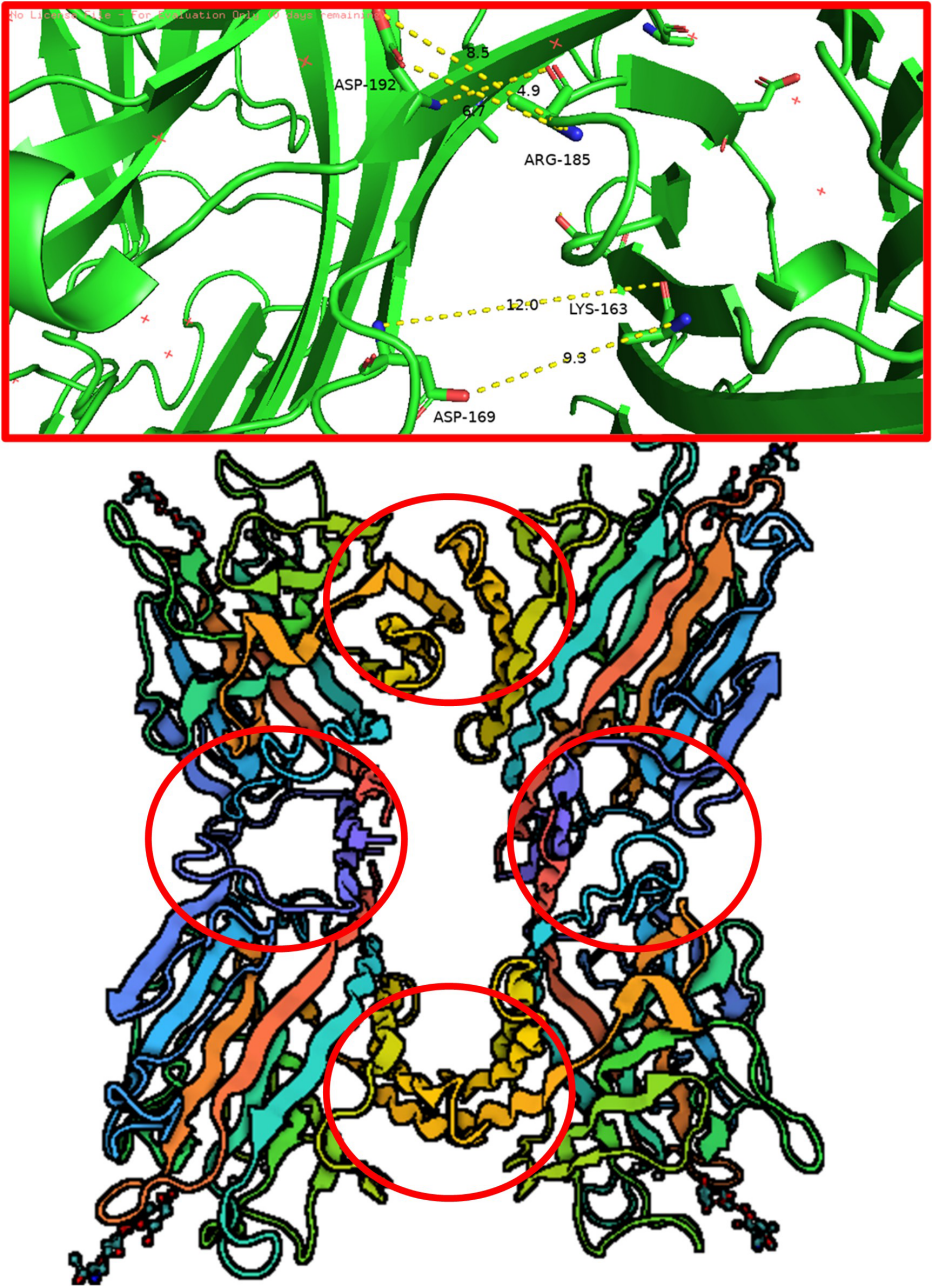
*In silico* representation of amino acid interactions maintaining the quaternary structure of soybean agglutinin, predicted using pyMOL.

The gene *Le1* (Glyma.02G012600) is responsible for lectin accumulation in soybean seeds. This trait is governed by the dominant allele (*LE*), whereas the recessive allele (*le*) carries an insertion element (Tgm1) that disrupts the gene’s reading frame, preventing lectin accumulation (Padalkar et al., 2023; Alves de Moraes et al., 2006; Vodkin et al., 1983). Expression analyses across different plant tissues indicate that *Le1* is predominantly expressed in seeds (**Figure 4**). Moreover, its promoter harbors a higher number of seed-specific motifs compared to other members of the PF03388 family (Chragh et al., 2015). Transcriptome profiling at various seed developmental stages further shows that *Le1* is the only family member expressed in this tissue, with peak mRNA levels occurring during mid-development (cotyledons of 100–200 mg) (Shamimuzzaman and Vodkin, 2018). Although *Le1* is specifically associated with seed lectin accumulation, other members of the lectin gene family remain expressed in vegetative tissues, such as leaves and roots, where they contribute to defense against herbivores and pathogens (Chrispeels and Raikhel, 1991; Lannoo and Van Damme, 2014; De Coninck and Van Damme, 2022).These observations suggest that introducing mutations in *Le1* is unlikely to affect other plant traits, which is corroborated by agronomic performance data (**Figure 13**), showing no differences between the edited plant and the non-edited cultivar in either plant height or grain yield in field conditions.

Regarding genetic transformation and generation of edited plants, soybean is considered a recalcitrant species. Despite adaptations that optimized transformation protocols, the efficiency remains low compared to species like rice, tobacco, and maize (Xu et al., 2020). In our work, the transformation efficiency was above to the average reported in the literature, which is around 3% (Xu et al., 2020). As for editing efficiency, two gRNAs were designed to target *Le1*, but only one showed editing in the events derived from stable transformation. Several factors might have contributed to this result, including the fact that the gRNAs were expressed under the AtU6 promoter from *Arabidopsis thaliana* in tandem, which may lead to competition for the transcription machinery, Additionally, the structure of the guide RNAs influences editing efficiency. By evaluating the structure of both guides, it is possible to identify that gRNA2 presented a more suitable configuration for Cas9 binding (**Supplementary Figure 1**). We observed that most edited events were obtained with gRNA 2, with an editing efficiency close to 10%. This result is similar to those reported in other studies using the *AtU6* promoter to drive gRNA expression (Sun et al., 2015; Du et al., 2016). Literature also indicates a significant increase in editing efficiency when the *GmU6–*10 promoter replaced *AtU6*-26 (Sun et al., 2015; Du et al., 2016; Di et al., 2019). Additionally, optimizing the Cas9 enzyme sequence can significantly affect editing efficiency (Michno et al., 2015, 2020; Cai et al., 2018).

Among the transgenic plants obtained, the AF12-13–1 plant exhibited a deletion of four amino acids at positions 388-391 (**Figure 7**). This mutation caused a frameshift and introduced a premature stop codon, resulting in a nonsense mutation. Bioinformatic analysis revealed that the truncated protein in AF12–13 consists of 159 amino acids and is classified as unstable. Soybean agglutinin is made up of four monomeric units that bind together to form a tetramer. The quaternary structure is stabilized by ionic interactions among the residues Asp-192, Arg-185, Asp-169, and Lys-163, which, positioned at different interfaces of the tetramer, collectively maintain the structural stability of the protein (**Figure 13**) (Sinha and Surolia, 2005; Sinha et al., 2007). The structural modification in the AF12-13–1 protein led to the loss of amino acids essential for maintaining the quaternary structure (**Figure 8**). Moreover, soybean agglutinin forms two dimers, which associate to form the tetrameric structure (dimers of dimers) (Halder et al., 2016). In the plant AF12-13-1, the amino acids mediating these dimer–dimer interactions are missing.

To perform its carbohydrate-binding activity and fulfill its biological function, the lectin must be properly glycosylated. The interaction between the carbohydrate and the protein requires the amino acids aspartate, asparagine, and an aromatic amino acid (Sharon and Lis, 2001). Mirkov and Chrispeels, 1993 showed that a mutation in Asn-128 of the leucoagglutinin (Pha-LEU) from *Phaseolus vulgaris* disrupts its carbohydrate-binding ability and abolishes the protein’s biological activity. Similarly, in *Pisum sativum*, van Eijsden et al., 1992 demonstrated that a mutation in Asn-125 of Psl eliminates the protein’s capacity to bind the monosaccharides glucose and mannose, which are essential for binding blood carbohydrates and hemagglutinating activity (Ma et al., 2011; Nabila et al., 2019; Tang et al., 2024).

The mutations introduced in the edited plant limited the lectin’s hemagglutination activity due to the absence of the protein’s quaternary structure in the AF12-13–1 plant (**Figure 13**). Therefore, the CRISPR/Cas9-induced mutation in the *Le1* gene of AF12-13–1 prevents full translation of the gene, resulting in a truncated protein. SDS-PAGE results support this finding, showing the protein’s absence in the mutated plant. In recessive *Le1* genotypes, the presence of the Tgm1 insertion element disrupts the protein’s reading frame and prevents its accumulation in the seed (Goldberg et al., 1983; Vodkin et al., 1983; Okamuro and Goldberg, 1992). The inactivation of soybean lectin, either using lectin-free genotypes, thermal processing, fermentation, or high-pressure treatment, has been consistently associated with improved animal performance and reduced intestinal damage caused by the deleterious effects of this soybean agglutinin (Pan et al., 2018; Han et al., 2023; Obeidat et al., 2025).

The generation advancement, monitored through molecular characterization by conventional PCR and sequencing, enabled the development of an plant free of the CRISPR/Cas machinery, which can be considered non-GMO under the Brazilian legislation. Overall, one of the major goals of soybean breeding is to eliminate or reduce antinutritional factors that impair animal growth performance, particularly in monogastric species (Douglas et al., 1999; Palacios et al., 2004; Palliyeguru et al., 2011; Hoffmann et al., 2019; Buzek et al., 2023; Nisley et al., 2025).

Recent studies have demonstrated that CRISPR-based genome editing can effectively inactivate genes responsible for these compounds, including the Kunitz trypsin inhibitor (KTI), the Bowman–Birk inhibitor (BBI), the raffinose family oligosaccharides, and phytic acid (Le et al., 2020; Cao et al., 2022; Song et al., 2022; Lin et al., 2023; Wang et al., 2023; Liu et al., 2025). In the near future, soybean lines carrying individual edits for these traits could be combined through conventional breeding, generating plants with reduced levels of multiple antinutritional factors. Conventional breeding efforts have already demonstrated the feasibility of pyramiding alleles associated with the natural absence of several antinutritional proteins. Classical crossing and marker-assisted selection have produced Triple Null and Penta Null soybean genotypes that combine recessive alleles for Kunitz trypsin inhibitor, soybean agglutinin, lipoxygenases, the 7S α′ subunit, and stachyose synthase (Choi et al., 2022; Schmidt et al., 2015). Together with our results, these genotypes confirm that eliminating multiple antinutritional traits does not compromise yield or seed composition. However, conventional approaches are time-consuming, require multiple generations, and depend on existing natural variation, highlighting the value of genome editing for achieving similar or broader outcomes with greater precision and efficiency.

In this context, CRISPR-based approaches have already achieved multigene improvements within a single generation. Multiplex editing of raffinose synthase genes (RS2 and RS3) has effectively reduced raffinose-family oligosaccharides without affecting seed viability or productivity (Cao et al., 2022; Lin et al., 2023), while simultaneous knockouts of Kunitz trypsin inhibitor and Bowman–Birk inhibitor genes have generated low-protease-inhibitor soybean lines with improved digestibility and feed performance (Wang et al., 2023; Liu et al., 2025). Beyond the specific knockout of Le1 achieved in this study, these advances illustrate a broader paradigm shift in crop biotechnology, transitioning from single-gene modification toward coordinated optimization of complex, nutritionally relevant traits.

Emerging CRISPR-derived tools, such as base and prime editing, further expand the potential for precise modulation of multiple loci related to nutritional composition, stress resilience, and yield stability (Li et al., 2023). Similar integrated strategies have been successfully applied in Brassica crops to balance glucosinolate biosynthesis and optimize both defense and nutritional quality (Cheng et al., 2024). Combining multiplex editing with genomic selection and metabolomic profiling is expected to accelerate the development of soybean cultivars that integrate enhanced feed value, sustainability, and robust agronomic performance (Chen et al., 2024).

Ultimately, soybean quality improvement should not focus solely on eliminating individual antinutritional factors, such as lectin, KTI, or oligosaccharides, but rather on achieving an optimized equilibrium between nutritional quality, resilience, and yield. This integrated perspective defines a forward-looking pathway for next-generation soybean breeding.

## 5 Conclusion

The *Le1* gene has been characterized as being exclusively expressed in seeds, and its knockout did not affect plant growth or yield;

Gene editing via CRISPR/Cas9 proved to be an efficient strategy for generating low-lectin varieties, producing a truncated protein that lost both its biological activity and the ability to form a quaternary structure;

Taken together, these results demonstrate that CRISPR/Cas9-mediated editing of seed antinutritional proteins, such as lectins, is an effective strategy to improve soybean grain quality for animal feed.

## Data Availability

The plasmid vector generated and used in this study has been deposited in the GenBank database (Accession number PX725410). The complete nucleotide sequence of the vector is also available in the **Supplementary Materials** of this article.
