# Supplementary material for: CRISPR/Cas9-mediated inactivation of the soybean agglutinin Le1 gene to improve grain quality
Source: Front Plant Sci. 2026 Jan 9;16:1737513. doi: 10.3389/fpls.2025.1737513 (PMC12829480; doi:10.3389/fpls.2025.1737513)
Supplement: Supplementary file 1 [file DataSheet1.docx]

Supplementary Material

**Supplementary table 1.** List of primes used for the detection of transgenics and absence of editing machinery.

| ID | Sequence 5'-3' | Tm (oC) | Amplicon (pb) |
| --- | --- | --- | --- |
| U6-Cloning-F | GTCGACGAATTCCTTCGTTGAACAACG | 55,34 | 564 |
| U6-Cloning-R | GGTACCGACAAAAAAAGCACCGACTC | 54,96 |  |
| GmLE1-F | ATGGCTACTTCAAAGTTGAAA | 52,34 | 858 |
| GmLE1-R | TTAGATGGCCTCATGCAAC | 53,93 |  |
| Bar-CRISPR-F1 | aaaggacagtagaaaaggaaga | 54,2 | 544 |
| Bar-CRISPR-R1 | aaggtagaagcagaaacttacc | 53,9 |  |
| Bar-CRISPR-F2 | tggaaggtaagtttctgctt | 54,3 | 776 |
| Bar-CRISPR-R2 | ccacgatttgacacattttt | 56 |  |
| Cas9-F1 | ccaacttcgataagaacctg | 55 | 845 |
| Cas9-R1 | ggagttcttctgtcccttct | 55 |  |
| Cas9-F6 | actccagaatgagaagctgt | 54 | 629 |
| Cas9-R6 | ccttgtagtcaccatacacg | 54 |  |


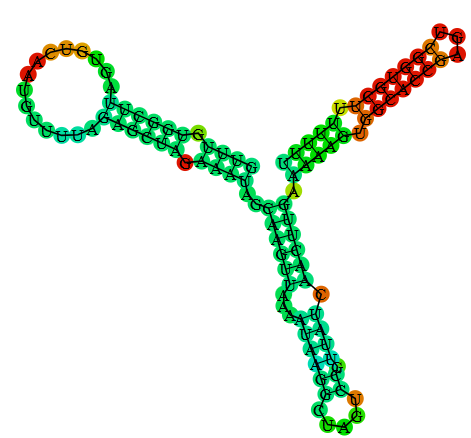


**Supplementary Figure 1.** gRNA 1 structure+Scaffold evaluated by RNA fold (http://rna.tbi.univie.ac.at//cgi-bin/RNAWebSuite/RNAfold.cgi)


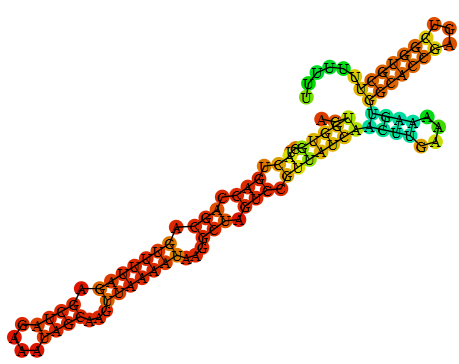


**Supplementary Figure 2.** gRNA 2 structure+Scaffold evaluated by RNA fold (<http://rna.tbi.univie.ac.at//cgi-bin/RNAWebSuite/RNAfold.cgi>)

**Supplementary Material 3.** Full Nucleotide Sequence of the C034p7ioR-35SCasWT-LE1 Plasmid

ggcaggatatattcaattgtaaatggcttcatgtccgggaaatctacatggatcagcaatgagtatgatggtcaatatggagaaaaagaaagagtaattaccaattttttttcaattcaaaaatgtagatgtccgcagcgttattataaaatgaaagtacattttgataaaacgacaaattacgatccgtcgtatttataggcgaaagcaataaacaaattattctaattcggaaatctttatttcgacgtgtctacattcacgtccaaatgggggcttagatgagaaacttcacgatcggctctagaacatggtggagcacgacactctcgtctactccaagaatatcaaagatacagtctcagaagaccagagggctattgagacttttcaacaaagggtaatatcgggaaacctcctcggattccattgcccagctatctgtcacttcatcgaaaggacagtagaaaaggaagatggcttctacaaatgccatcattgcgataaaggaaaggctatcgttcaagatgcctctaccgacagtggtcccaaagatggacccccacccacgaggaacatcgtggaaaaagaagacgttccaaccacgtcttcaaagcaagtggattgatgtgatatctccactgacgtaagggatgacgcacaatcccactatccttcgcaagacccttcctctatataaggaagttcatttcatttggagaggacctcgagtcctccataactatgggccccgagcgcaggcccgccgacatccgccgtgccaccgaggccgacatgccagccgtctgcaccatcgtgaaccactacatcgagacctccacggtcaacttccgcacagagccgcaggagcctcaggagtggaccgacgacctcgtgaggctgcgcgagcgctacccctggctcgtcgctgaggtggatggcgaggtcgccggcatcgcctacgcgggcccctggaaggtaagtttctgcttctacctttgatatatatataataattatcattaattagtagtaatataatatttcaaatatttttttcaaaataaaagaatgtagtatatagcaattgcttttctgtagtttataagtgtgtatattttaatttataacttttctaatatatgaccaaaatttgttgatgtgcaggcacgcaacgcctacgactggacggcggagagcaccgtctacgtgtccccacgccaccagcgcaccggcctgggctccacgctctacacccacctgctcaagagcctggaggcccagggcttcaagtccgtggtcgctgtgatcggcctccccaacgacccgagcgtccgtatgcacgaggccctcggctacgcgccccgcgggatgctgagggccgcgggcttcaagcacggcaactggcacgacgtgggcttctggcagctcgacttctccctgcctgtcccaccccgcccggtgctccccgtcaccgaaatctgaacgcgttcgagtattatggcattgggaaaactgtttttcttgtaccatttgttgtgcttgtaatttactgtgttttttattcggttttcgctatcgaactgtgaaatggaaatggatggagaagagttaatgaatgatatggtccttttgttcattctcaaattaatattatttgttttttctcttatttgttgtgtgttgaatttgaaattataagagatatgcaaacattttgttttgagtaaaaatgtgtcaaatcgtggcctctaatgaccgaagttaatatgaggagtaaaacactgaagcctgcaggcatgcaagctgatccactagaggccatggcggccgcactagatcaacatggtggagcacgacactctcgtctactccaagaatatcaaagatacagtctcagaagaccagagggctattgagacttttcaacaaagggtaatatcgggaaacctcctcggattccattgcccagctatctgtcacttcatcgaaaggacagtagaaaaggaagatggcttctacaaatgccatcattgcgataaaggaaaggctatcgttcaagatgcctctaccgacagtggtcccaaagatggacccccacccacgaggaacatcgtggaaaaagaagacgttccaaccacgtcttcaaagcaagtggattgatgtgatatctccactgacgtaagggatgacgcacaatcccactatccttcgcaagacccttcctctatataaggaagttcatttcatttggagaggacactagttggatcctccataactatggcccccaagaagaagcgcaaagtgggaatccacggagctccagacaagaagtacagcattggtctggacatcgggaccaactccgtcggctgggccgttatcaccgatgagtacaaagtgccttctaagaagttcaaagtcctcggaaacactgacaggcacagcatcaagaagaacttgattggcgcccttctcttcgactccggcgaaaccgctgaggccacccgtctgaagcgcaccgccagaaggcgctacacccgtcgcaagaataggatctgctacctccaggagatcttcagcaacgagatggccaaagtggacgattccttctttcaccgtttggaggagagcttccttgtcgaggaggacaagaagcacgagcgccatcccatcttcggaaacattgttgacgaggtggcttaccacgagaagtatccaactatctaccacctcagaaagaagctggtggactccaccgacaaggccgatttgaggctcatctaccttgccctcgctcacatgatcaagttccgcggtcacttcctgattgagggcgacctcaaccccgacaactctgacgtcgataagttgttcatccaactggtccagacctacaaccagcttttcgaggagaatcctatcaacgcctccggggttgacgccaaggctattctcagcgcccgtctgtccaagagccgcaggcttgagaacttgatcgcccagctcccaggagagaagaagaacggcctcttcggaaacctgatcgctctctccttgggccttacccctaacttcaagtctaattttgacctcgccgaggacgccaagctgcaactcagcaaggacacctacgatgacgacttggacaacctgctcgcccagatcggcgatcagtacgctgaccttttcttggccgccaagaacctctccgacgctattctgctctccgacatccttcgcgtgaacactgagatcaccaaggccccactgagcgcctccatgatcaagcgttacgacgaacaccaccaagatttgaccctcctcaaggctcttgtcagacagcagctgcccgagaagtacaaggagattttcttcgaccagagcaagaacggatacgccggttacatcgacggcggggcctcccaagaggagttctacaagttcatcaagcccatcttggagaagatggacggcaccgaagagctgctcgtgaagctcaaccgcgaggatcttttgaggaagcagcgtaccttcgacaacggatctattccccaccagatccatctcggcgagctgcacgctatcctccgcaggcaagaggacttctatccattccttaaggacaatcgcgagaagattgaaaagatcctcacttttagaatcccttactacgtcggacccttggcccgtggcaacagccgcttcgcctggatgaccaggaagtccgaggaaaccatcaccccatggaacttcgaggaggtggttgacaagggggccagcgctcagtccttcattgagcgcatgaccaacttcgataagaacctgcccaacgagaaagtcctcccaaagcactcccttctgtacgagtacttcactgtctacaatgagttgaccaaagtgaagtacgtgaccgagggtatgcgtaagcctgccttcctcagcggcgagcagaagaaggccatcgttgacctcctgttcaagaccaacaggaaagtcaccgtgaagcagcttaaggaggactacttcaagaagatcgagtgctttgactctgtcgagatcagcggagtggaggatcgcttcaacgcttccttgggcacttaccacgacctcctgaagattatcaaggacaaggacttcctcgacaacgaggagaacgaagatatcttggaggacatcgttcttaccctcaccctgttcgaggacagagagatgattgaggaacgcctcaagacctacgcccacttgttcgacgataaagtcatgaagcaacttaagcgtaggcgctacaccggatggggcaggctgtcccgtaagctcatcaacggtatccgcgacaagcagagcggcaagactattctcgacttcttgaagtccgacggattcgccaatcgcaacttcatgcagctgatccacgacgattctcttaccttcaaggaggacatccaaaaggctcaagtgtccggccagggcgacagcctccacgagcacatcgccaacctggccgggagccccgctattaagaagggaatcctccagaccgtcaaggtggttgacgagttggtcaaggtgatgggcagacataagccagagaacatcgtcatcgagatggccagggaaaaccagaccacccagaagggacagaagaactcccgtgagcgcatgaagaggattgaggagggcatcaaggagcttggttcccagatcctcaaggagcaccccgtggaaaacacccaactccagaatgagaagctgtatttgtactaccttcagaacgggcgcgatatgtacgttgaccaagagctggacatcaaccgcctcagcgactacgacgtggatcacattgtccctcagtcctttctcaaggacgactctatcgacaacaaggtgttgactagaagcgataagaaccgcggcaagtccgacaacgtcccatccgaggaggtggtcaagaagatgaagaattactggaggcagctgcttaacgccaagctcatcacccagcgcaagttcgacaacttgaccaaggccgagcgtggaggcctcagcgaactggacaaggctggattcatcaagaggcaacttgttgaaacccgccagattaccaagcacgtggcccagatcctcgactcccgtatgaacactaagtacgatgagaacgacaagctgatccgcgaggtcaaagtgattaccctcaagagcaagctcgtgtctgacttcagaaaggacttccaattctacaaggttagggagatcaacaactaccaccacgcccacgatgcttaccttaatgccgtggtcggcaccgccttgatcaagaagtaccccaagctggagtccgagttcgtgtatggtgactacaaggtctacgacgttcgcaagatgatcgctaagagcgagcaggagattggcaaggccaccgccaagtacttcttctactccaacatcatgaacttctttaagaccgagatcactttggctaacggggagatccgtaagcgccctctcattgaaaccaacggagaaaccggcgagatcgtgtgggacaagggcagggacttcgccaccgtcagaaaagtgctgagcatgccccaagtcaacatcgtgaagaaaaccgaggttcagactggaggcttctccaaggagtctatcctcccaaagcgcaattccgataagttgattgcccgtaagaaggactgggaccccaagaagtacggtggattcgacagcccaaccgtcgcctactccgtgcttgtcgtggctaaagttgagaagggcaagagcaagaagctcaagtccgtcaaggagctgctcgggatcaccatcatggagcgcagctccttcgagaagaaccctattgatttccttgaggccaagggctacaaggaagtgaagaaggacttgatcatcaagctccccaagtactctctgttcgagcttgagaacggaaggaagcgtatgctcgcctccgctggcgagctgcaaaagggaaacgagttggccctcccaagcaagtacgtcaacttcctgtacctcgcctcccactatgagaagctcaagggcagccccgaggacaacgaacagaagcagttgtttgtggagcagcataagcactaccttgacgagatcattgagcagatcagcgagttctccaagcgcgttatcctggctgacgccaatctcgataaagtcctttctgcctacaacaagcacagggacaagcctatcagagagcaggctgaaaacattatccacttgttcaccctcaccaacctgggtgccccagccgccttcaagtacttcgacactaccatcgaccgcaagcgttacacctccaccaaggaagtgctcgatgctacccttatccaccagagcattactgggttgtacgaaaccaggatcgacctgtcccaactcggcggagacaagcgccccgccgccaccaagaaggctggccaggccaagaagaagaagtaacccgggatcgttcaaacatttggcaataaagtttcttaagattgaatcctgttgccggtcttgcgatgattatcatataatttctgttgaattacgttaagcatgtaataattaacatgtaatgcatgacgttatttatgagatgggtttttatgattagagtcccgcaattatacatttaatacgcgatagaaaacaaaatatagcgcgcaaactaggataaattatcgcgcgcggtgtcatctatgttactagatcgctcgaattccttcgttgaacaacggaaactcgacttgccttccgcacaatacatcatttcttcttagctttttttcttcttcttcgttcatacagtttttttttgtttatcagcttacattttcttgaaccgtagctttcgttttcttctttttaactttccattcggagtttttgtatcttgtttcatagtttgtcccaggattagaatgattaggcatcgaaccttcaagaatttgattgaataaaacatcttcattcttaagatatgaagataatcttcaaaaggcccctgggaatctgaaagaagagaagcaggcccatttatatgggaaagaacaatagtatttcttatataggcccatttaagttgaaaacaatcttcaaaagtcccacatcgcttagataagaaaacgaagctgagtttatatacagctagagtcgaagtagtgattgagacgttcgaagcttctgtcgtctcagttttagagctagaaatagcaagttaaaataaggctagtccgttatcaacttgaaaaagtggcaccgagtcggtgctttttttgaattccttcgttgaacaacggaaactcgacttgccttccgcacaatacatcatttcttcttagctttttttcttcttcttcgttcatacagtttttttttgtttatcagcttacattttcttgaaccgtagctttcgttttcttctttttaactttccattcggagtttttgtatcttgtttcatagtttgtcccaggattagaatgattaggcatcgaaccttcaagaatttgattgaataaaacatcttcattcttaagatatgaagataatcttcaaaaggcccctgggaatctgaaagaagagaagcaggcccatttatatgggaaagaacaatagtatttcttatataggcccatttaagttgaaaacaatcttcaaaagtcccacatcgcttagataagaaaacgaagctgagtttatatacagctagagtcgaagtagtgattgagacgttcgaagcttctgtcgtctcagttttagagctagaaatagcaagttaaaataaggctagtccgttatcaacttgaaaaagtggcaccgagtcggtgctttttttgtcgacaggccttaagggccagatcttgggcccggtacccgatcagattgtcgtttcccgccttcggtttaaactatcagtgtttgacaggatatattggcgggtaaacctaagagaaaagagcgtttattagaataatcggatatttaaaagggcgtgaaaaggtttatccgttcgtccatttgtatgtgcatgccaaccacagggttcccctcgggagtgcttggcattccgtgcgataatgacttctgttcaaccacccaaacgtcggaaagcctgacgacggagcagcattccaaaaagatcccttggctcgtctgggtcggctagaaggtcgagtgggctgctgtggcttgatccctcaacgcggtcgcggacgtagcgcagcgccgaaaaatcctcgatcgcaaatccgacgctgtcgaaaagcgtgatctgcttgtcgctctttcggccgacgtcctggccagtcatcacgcgccaaagttccgtcacaggatgatctggcgcgagttgctggatctcgccttcaatccgggtctgtggcgggaactccacgaaaatatccgaacgcagcaagatatcgcggtgcatctcggtcttgcctgggcagtcgccgccgacgccgttgatgtggacgccgaaaaggatctaggtgaagatcctttttgataatctcatgaccaaaatcccttaacgtgagttttcgttccactgagcgtcagaccccgtagaaaagatcaaaggatcttcttgagatcctttttttctgcgcgtaatctgctgcttgcaaacaaaaaaaccaccgctaccagcggtggtttgtttgccggatcaagagctaccaactctttttccgaaggtaactggcttcagcagagcgcagataccaaatactgttcttctagtgtagccgtagttaggccaccacttcaagaactctgtagcaccgcctacatacctcgctctgctaatcctgttaccagtggctgctgccagtggcgataagtcgtgtcttaccgggttggactcaagacgatagttaccggataaggcgcagcggtcgggctgaacggggggttcgtgcacacagcccagcttggagcgaacgacctacaccgaactgagatacctacagcgtgagctatgagaaagcgccacgcttcccgaagggagaaaggcggacaggtatccggtaagcggcagggtcggaacaggagagcgcacgagggagcttccagggggaaacgcctggtatctttatagtcctgtcgggtttcgccacctctgacttgagcgtcgatttttgtgatgctcgtcaggggggcggagcctatggaaaaacgccagcaacgcggcctttttacggttcctggccttttgctggccttttgctcacatgttctttcctgcgttatcccctgattctgtggataaccgattaccgcctttgagtgagctgataccgctcgccgcagccgaacgaccgagcgcagcgagtcagtgagcgaggaagcggaagagcgcctgatgcggtattttctccttacgcatctgtgcggtatttcacaccgcatatggtgcactctcagtacaatctgctctgatgccgcatagttaagccagtaaccgtgcggctgcatgaaatcctggccggtttgtctgatgccaagctcgcggcctggccggcgagcttggccgctgaagaaaccgagcgccgccgtctaaaaaggtgatgtgtatttgagtaaaacagcttgcgtcatgcggtcgctgcgtatatgatgcgatgagtaaataaacaaatacgcaaggggaacgcatgaaggttatcgctgtacttaaccagaaaggcgggtcaggcaagacgaccatcgcaacccatctagcccgcgccctgcaactcgccggggccgatgttctgttagtcgattccgatccccagggcagtgcccgcgattgggcggccgtgcgggaagatcaaccgctaaccgttgtcggcatcgaccgcccgacgattgaccgcgacgtgaaggccatcggccggcgcgacttcgtagtgatcgacggagcgccccaggcggcggacttggctgtgtccgcgatcaaggcagccgacttcgtgctgattccggtgcagccaagcccttacgacatatgggccaccgccgacctggtggagctggttaagcagcgcattgaggtcacggatggaaggctacaagcggcctttgtcgtgtcgcgggcgatcaaaggcacgcgcatcggcggtgaggttgccgaggcgctggccgggtacgagctgcccattcttgagtcccgtatcacgcagcgcgtgagctacccaggcactgccgccgccggcacaaccgttcttgaatcagaacccgagggcgacgctgcccgcgaggtccaggcgctggccgctgaaattaaatcaaaactcatttgagttaatgaggtaaagagaaaatgagcaaaagcacaaacacgctaagtgccggccgtccgagcgcacgcagcagcaaggctgcaacgttggccagcctggcagacacgccagccatgaagcgggtcaactttcagttgccggcggaggatcacaccaagctgaagatgtacgcggtacgccaaggcaagaccattaccgagctgctatctgaatacatcgcgcagctaccagagtaaatgagcaaatgaataaatgagtagatgaattttagcggctaaaggaggcggcatggaaaatcaagaacaaccaggcaccgacgccgtggaatgccccatgtgtggaggaacgggcggttggccaggcgtaagcggctgggttgtctgccggccctgcaatggcactggaacccccaagcccgaggaatcggcgtgagcggtcgcaaaccatccggcccggtacaaatcggcgcggcgctgggtgatgacctggtggagaagttgaaggcggcgcaggccgcccagcggcaacgcatcgaggcagaagcacgccccggtgaatcgtggcaagcggccgctgatcgaatccgcaaagaatcccggcaaccgccggcagccggtgcgccgtcgattaggaagccgcccaagggcgacgagcaaccagattttttcgttccgatgctctatgacgtgggcacccgcgatagtcgcagcatcatggacgtggccgttttccgtctgtcgaagcgtgaccgacgagctggcgaggtgatccgctacgagcttccagacgggcacgtagaggtttccgcagggccggccggcatggcgagtgtgtgggattacgacctggtactgatggcggtttcccatctaaccgaatccatgaaccgataccgggaagggaagggagacaagcccggccgcgtgttccgtccacacgttgcggacgtactcaagttctgccggcgagccgatggcggaaagcagaaagacgacctggtagaaacctgcattcggttaaacaccacgcacgttgccatgcagcgtacgaagaaggccaagaacggccgcctggtgacggtatccgagggtgaagccttgattagccgctacaagatcgtaaagagcgaaaccgggcggccggagtacatcgagatcgagctagctgattggatgtaccgcgagatcacagaaggcaagaacccggacgtgctgacggttcaccccgattactttttgatcgatcccggcatcggccgttttctctaccgcctggcacgccgcgccgcaggcaaggcagaagccagatggttgttcaagacgatctacgaacgcagtggcagcgccggagagttcaagaagttctgtttcaccgtgcgcaagctgatcgggtcaaatgacctgccggagtacgatttgaaggaggaggcggggcaggctggcccgatcctagtcatgcgctaccgcaacctgatcgagggcgaagcatccgccggttcctaatgtacggagcagatgctagggcaaattgccctagcaggggaaaaaggtcgaaaaggtctctttcctgtggatagcacgtacattgggaacccaaagccgtacattgggaaccggaacccgtacattgggaacccaaagccgtacattgggaaccggtcacacatgtaagtgactgatataaaagagaaaaaaggcgatttttccgcctaaaactctttaaaacttattaaaactcttaaaacccgcctggcctgtgcataactgtctggccagcgcacagccgaagagctgcaaaaagcgcctacccttcggtcgctgcgctccctacgccccgccgcttcgcgtcggcctatcgcggccgctggccgctcaaaaatggctggcctacggccaggcaatctaccagggcgcggacaagccgcgccgtcgccactcgaccgccggcgcccacatcaaggcaccggtgggtatgcctgacgatgcgtggagaccgaaaccttgcgctcgttcgccagccaggacagaaatgcctcgacttcgctgctgcccaaggttgccgggtgacgcacaccgtggaaacggatgaaggcacgaacccagtggacataagcctgttcggttcgtaagctgtaatgcaagtagcgtatgcgctcacgcaactggtccagaaccttgaccgaacgcagcggtggtaacggcgcagtggcggttttcatggcttgttatgactgtttttttggggtacagtctatgcctcgggcatccaagcagcaagcgcgttacgccgtgggtcgatgtttgatgttatggagcagcaacgatgttacgcagcagggcagtcgccctaaaacaaagttaaacatcatgagggaagcggtgatcgccgaagtatcgactcaactatcagaggtagttggcgtcatcgagcgccatctcgaaccgacgttgctggccgtacatttgtacggctccgcagtggatggcggcctgaagccacacagtgatattgatttgctggttacggtgaccgtaaggcttgatgaaacaacgcggcgagctttgatcaacgaccttttggaaacttcggcttcccctggagagagcgagattctccgcgctgtagaagtcaccattgttgtgcacgacgacatcattccgtggcgttatccagctaagcgcgaactgcaatttggagaatggcagcgcaatgacattcttgcaggtatcttcgagccagccacgatcgacattgatctggctatcttgctgacaaaagcaagagaacatagcgttgccttggtaggtccagcggcggaggaactctttgatccggttcctgaacaggatctatttgaggcgctaaatgaaaccttaacgctatggaactcgccgcccgactgggctggcgatgagcgaaatgtagtgcttacgttgtcccgcatttggtacagcgcagtaaccggcaaaatcgcgccgaaggatgtcgctgccgactgggcaatggagcgcctgccggcccagtatcagcccgtcatacttgaagctagacaggcttatcttggacaagaagaagatcgcttggcctcgcgcgcagatcagttggaagaatttgtccactacgtgaaaggcgagatcaccaaggtagtcggcaaataatgtctaacaattcgttcaagccgacgccgcttcgcggcgcggcttaactcaagcgttagatgcactaagcacataattgctcacagccaaactatcaggtcaagtctgcttttattatttttaagcgtgcataataagccctacacaaattgggagatatatcatgaaaggctggctttttcttgttatcgcaatagttggcgaagtaatcgcaacatagcttgcttggtcgttccgcgtgaacgtcggctcgattgtacctgcgttcaaatactttgcgatcgtgttgcgcgcctgcccggtgcgtcggctgatctcacggatcgactgcttctctcgcaacgccatccgacggatgatgtttaaaagtcccatgtggatcactccgttgccccgtcgctcaccgtgttggggggaaggtgcacatggctcagttctcaatggaaattatctgcctaaccggctcagttctgcgtagaaaccaacatgcaagctccaccgggtgcaaagcggcagcggc

CaMV Promoter

Bar Gene

STLS intron

E9 terminator

Cas9WT

NLS

NOS-T

AtU6 promoter

Scaffold
